# Sex-specific recombination patterns predict parent of origin for recurrent genomic disorders

**DOI:** 10.1186/s12920-021-00999-8

**Published:** 2021-06-09

**Authors:** Trenell J. Mosley, H. Richard Johnston, David J. Cutler, Michael E. Zwick, Jennifer G. Mulle

**Affiliations:** 1 Graduate Program in Genetics and Molecular Biology, Laney Graduate School, Emory University, 201 Dowman Drive, Atlanta, GA 30322 USA; 2 Department of Human Genetics, Emory University School of Medicine, 615 Michael Street, Whitehead Building Suite 300, Atlanta, GA 30322 USA; 3 Emory Integrated Computational Core, Emory University, 101 Woodruff Circle, Atlanta, GA 30322 USA; 4 Department of Pediatrics, Emory University School of Medicine, 2015 Uppergate Drive, Atlanta, GA 30322 USA; 5 Department of Epidemiology, Rollins School of Public Health, Emory University, 1518 Clifton Road NE, Atlanta, GA 30322 USA

**Keywords:** Copy number variants, Meiotic recombination, Parent of origin, 3q29 deletion

## Abstract

**Background:**

Structural rearrangements of the genome, which generally occur during meiosis and result in large-scale (> 1 kb) copy number variants (CNV; deletions or duplications ≥ 1 kb), underlie genomic disorders. Recurrent pathogenic CNVs harbor similar breakpoints in multiple unrelated individuals and are primarily formed via non-allelic homologous recombination (NAHR). Several pathogenic NAHR-mediated recurrent CNV loci demonstrate biases for parental origin of de novo CNVs. However, the mechanism underlying these biases is not well understood.

**Methods:**

We performed a systematic, comprehensive literature search to curate parent of origin data for multiple pathogenic CNV loci. Using a regression framework, we assessed the relationship between parental CNV origin and the male to female recombination rate ratio.

**Results:**

We demonstrate significant association between sex-specific differences in meiotic recombination and parental origin biases at these loci (*p* = 1.07 × 10^–14^).

**Conclusions:**

Our results suggest that parental origin of CNVs is largely influenced by sex-specific recombination rates and highlight the need to consider these differences when investigating mechanisms that cause structural variation.

**Supplementary Information:**

The online version contains supplementary material available at 10.1186/s12920-021-00999-8.

## Background

Genomic disorders are caused by pathological structural variation in the human genome usually arising de novo during parental meiosis [1–4]. The most common pathogenic variety of these rearrangements are copy number variants (CNVs), i.e. a deletion or duplication of > 1 kb of genetic material [3, 5, 6]. The clinical phenotypes of genomic disorders are varied. They include congenital dysmorphisms, neurodevelopmental, neurodegenerative, and neuropsychiatric manifestations, and even more common complex phenotypes such as obesity and hypertension [7–12]. CNVs have been observed in 10% of sporadic cases of autism [13, 14], 15% of schizophrenia cases [15, 16], and 16% of cases of intellectual disability [17]. These and other associations highlight the importance of structural variation to human health and the need to understand the factors influencing how they arise.

There is an intense interest in understanding the mechanisms by which CNVs form [18, 19]. In several regions of the genome, de novo CNVs with approximately the same breakpoints recur in independent meioses (recurrent CNVs) [1, 20]. The presence of segmental duplications flanking these intervals is a hallmark feature of recurrent CNVs. It is hypothesized that misalignment and subsequent recombination between non-allelic low copy repeat (LCR) segments within the segmental duplication regions is the formative event giving rise to the CNV [21, 22], so-called non-allelic homologous recombination (NAHR). Risk factors that may favor NAHR have been investigated and include sequence composition and orientation of the LCRs themselves [21, 23] as well as the presence of inversions at the locus [24, 25].

Parental sex bias for the origin of recurrent de novo CNVs remains unexplained. De novo deletions at the 16p11.2 and 17q11.2 loci are more likely to arise on maternally inherited chromosomes [26–29]. Deletions at the 22q11.2 locus show a slight maternal bias as well [30]. In contrast, deletions at the 5q35.3 locus (Sotos syndrome [MIM: 117550]) display a paternal origin bias [31, 32]. Deletions at the 7q11.23 locus (Williams syndrome [MIM: 194050]) do not show a bias in parental origin [24]. While it has been suggested that sex-specific recombination rates might influence sex biases in NAHR [26], this hypothesis has not been formally tested.

The majority of recurrent CNVs are thought to form during meiosis when homologous chromosomes align and synapse during prophase I [33]. It is well established that meiosis differs significantly between males and females. In males, spermatagonia continuously divide and complete meiosis throughout postpubescent life with all four products of meiosis resulting in gametes. In contrast, in human females, oogonia are established in fetal life and enter into an extended period of prolonged stasis in prophase I of meiosis until they complete meiosis upon ovulation and fertilization [34]. Additionally, in female meiosis, only one of four products of meiosis result in a gamete. Sexual dimorphism in meiosis extends to the patterns and processes of recombination during meiosis [33]. Here we seek to ask whether local sex-specific rates in meiotic recombination can predict the parental bias for the origin of recurrent de novo CNVs.

## Methods

### Parent of origin determination

#### Literature search and parental origin data curation

For this analysis, we considered the 55 known genomic disorder CNV loci described in Coe et al. [7]. A locus was eligible for inclusion in the current analysis if it is flanked by LCRs, i.e. mediated by NAHR, and not imprinted (n = 38 eligible loci). For each of these 38 loci, we performed a systematic PubMed search to identify published data on parental origin. Studies were admitted to this paper’s analysis when the following criteria were met: (1) the study detailed parent of origin data for one of the 38 eligible NAHR-mediated loci as designated by Coe et al. [7], (2) the authors of the study interrogated the entire canonical CNV interval to confirm the presence of a deletion or duplication in the patients, (3) the authors determined the investigated CNVs were de novo, and (4) the study clearly treated monozygotic twins as one meiotic event and not two (Additional file 1: Supplemental Methods, Additional file 2: Table S1, and Additional file 3: Table S2). The literature search led to a manual review of 1268 papers, out of which we identified 77 manuscripts across 24 loci with suitable data for analysis: 1q21.1 [35–39], 1q21.1 TAR [40], 2q13 [37], 3q29 [37–42], 5q35 [31, 32], 7q11.23 [24, 40, 43–54], 8p23.1 [55, 56], 11q13.2q13.4 [57], 15q13.3 [38, 40, 58], 15q24 (AC, AD, BD, and BE intervals) [59–64], 15q25.2 [65–67],16p11.2 [26, 37, 40, 68–70], distal 16p11.2 [37, 38, 70], 16p11.2p12.1 [71], 16p31.11 [37], 17p11.2 [72–76], 17q11.2 [28, 29, 77], 17q12 [37, 38, 78], 17q21.31 [19, 25, 67, 79–84], 17q23.1q23.2 [69, 85] and 22q11.2 [30, 43, 53, 86–102] (Table 1). For the remaining 14 loci, no published parent of origin data could be identified. At the 3q29 locus, we generated new data to determine the parent of origin for de novo events (http://genome.emory.edu/3q29/).

**Table 1:** Summary of CNV loci included in literature search and curated studies

Locus	MIM number	# Studies included^a^	Study references
1q21.1	612474/612475	5	[35–39]
1q21.1 TAR	274000	1	[40]
2q11.2	**–**	0	**–**
2q11.2q13	**–**	0	**–**
2q13	**–**	1	[37]
3q29	609425/611936	6	[37–42]
5q35	117550	2	[31, 32]
7q11.23	194050/609757	14	[24, 40, 43–54]
7q11.23 distal	613729	0	**–**
7q11.23 proximal	**–**	0	**–**
8p23.1	**–**	2	[55, 56]
10q23	612242	0	**–**
11q13.2q13.4	**–**	1	[57]
15q11.2	615656	0^b^	**–**
15q13.3	612001	3	[38, 40, 58]
15q24^c^	**–**	6	[59–64]
15q25.2	614294	3	[65–67]
15q25.2 (Cooper)	**–**	0	**–**
16p11.2	611913/614671	6	[26, 37, 40, 68–70]
16p11.2 distal	613444	3	[37, 38, 70]
16p11.2p12.2	**–**	0	**–**
16p11.2p12.1	**–**	1	[71]
16p12.1	136570	0	**–**
16p13.11	**–**	1	[37]
17p11.2	182290/610883	5	[72–76]
17p11.2p12^d^	118220/162500	0	**–**
17q11.2	613675/618874	3	[28, 29, 77]
17q12	614526/614527	3	[37, 38, 78]
17q21.31	610443/613533	9	[19, 25, 67, 79–84]
17q23	**–**	0	**–**
17q23.1q23.2	613355/613618	2	[69, 85]
22q11.2	188400/192430	20	[30, 43, 53, 86–102]
22q11.2 distal	611867	0	**–**

^a^Independent studies from which the parent of origin data for the current analysis were obtained. Studies may be repeated between loci

^b^Recombination rates could not be calculated for 15q11.2 as the breakpoints were outside the range of recombination maps

^c^15q24 locus is represented as 6 different intervals in Coe et al. [7]

^d^17p11.2p12 excluded due to inconsistencies in the mechanism of formation. See Additional file 1: Supplemental Methods

#### Determination of parental origin for 3q29 deletion

##### Study subject recruitment

This study was approved by Emory University’s Institutional Review Board (IRB00064133). Individuals with a clinically confirmed diagnosis of 3q29 deletion were ascertained through the internet-based 3q29 registry (https://3q29deletion.patientcrossroads.org/) as previously described [103]. Blood samples were obtained from 14 families. SNP genotyping was performed on 12 of the 14 families (10 full trios, 2 mother–child pairs) using the Illumina GSA-24 v 3.0 array. For 2 full trios (6 samples), parent of origin was determined from whole-genome sequence data on Illumina's NovaSeq 6000 platform. Quality control was performed with PLINK 1.9 [104] and our custom pipeline (Additional file 1: Supplemental Methods).

##### Parental origin analysis

Parental origin of the 3q29 deletion was determined for all 14 families using PLINK 1.9 [104]. SNPs located within the 3q29 deletion region (chr3:196029182–197617792; hg38) were isolated for analysis and the pattern of Mendelian errors (MEs) were analyzed. The parent with the most MEs was considered the parent of origin for the 3q29 deletion (Additional file 1: Supplemental Methods). The mean age of fathers in our 3q29 cohort was collected from self-reported data in conjunction with the Emory University 3q29 project (http://genome.emory.edu/3q29/) and compared to the U.S. average (NCHS; https://www.cdc.gov/nchs/index.htm) via a two-tailed two-sample t-test using R [105].

### Calculation of recombination rates and ratios

Chromosome male and female recombination rates (cM/Mb) were obtained from the deCODE sex-specific maps, which are based on over 4.5 million crossover recombination events from 126,427 meioses, with an average resolution of 682 base pairs [106]. The recombination rate (cM/Mb) data from deCODE is publicly available as recombination rates calculated for a physical genomic interval bounded by two SNP markers (Additional file 1: Supplemental Methods). Therefore, for our calculation of the average male and female recombination rates, each bounded recombination rate was weighted by the total number of base pairs contained within the respective SNP marker interval. Weighted rates were then averaged across the CNV interval for males and females, separately. The ratio of the weighted average male and female recombination rates was then calculated for each CNV interval by dividing the weighted average male recombination rate by the weighted average female recombination rate (Additional file 1: Figure S1). To account for slight differences in the recombination rate ratios calculated for the different LCR22 intervals at the 22q11.2 locus we used an adjusted recombination rate ratio composed of the weighted recombination rate ratios calculated for each LCR22 interval. Weights were based on the estimated population prevalence of the different 22q11.2 deletion intervals (Additional file 1: Table S3) [107].

### Logistic regression analysis

Parental origin data was curated for CNVs at the 24 CNV loci from 77 independent studies; only independent samples were included in the analysis (duplicate or overlapping samples were removed). For each CNV locus, the male to female recombination rate ratio was calculated as described above. A logistic regression model was fitted to the data with the log_e_-transformed male to female recombination rate ratio as the predictor and parental origin (paternal vs. maternal) as the response variable. We performed a secondary analysis stratified by deletions and duplications. See Table 2 and Additional file 4: Table S4 for the data calculated and used in the logistic regression analyses.

**Table 2:** Summary of genomic disorder loci CNVs recombination calculations

Locus	CNV type	BED coordinates [7]	# Samples (%)	M:F origin counts^a^	Del/dup counts	Avg. male recombination rate [106]^b^	Avg. female recombination rate [106]^b^	Log_e_ M:F recombination ratio [106]^c^
1q21.1	Del/Dup	chr1:147101794–147921262	9 (0.46%)	6:3	7/2	0.12331689	0.50839541	− 1.416626
1q21.1 TAR	Del	chr1:145686999–146048495	1 (0.05%)	1:0	1/0	0.15712388	0.77814863	− 1.599883
2q13	Dup	chr2:110625954–112335952	1 (0.05%)	1:0	0/1	0.44854539	1.64377881	− 1.298743
3q29	Del	chr3:195988732–197628732	22 (1.11%)	21:1	22/0	3.1305211	0.27775988	2.422197
5q35	Del	chr5:176290391–177630393	41 (2.07%)	36:5	41/0	1.29955355	0.97941355	0.282822
7q11.23	Del/Dup	chr7:73328061–74727726	618 (31.26%)	296:322	598/20	0.49353554	1.92657023	− 1.361890
8p23.1	Del/Dup	chr8:8235068–12035082	3 (0.15%)	1:2	1/2	0.67201752	1.81857951	− 0.995527
11q13.2q13.4	Del	chr11:67985953–71571306	1 (0.05%)	0:1	1/0	0.8431765	2.23501635	− 0.974828
15q13.3	Del/Dup	chr15:30840505–32190507	6 (0.30%)	5:1	5/1	1.63640726	1.89901039	− 0.148828
15q24 AC	Del	chr15:72670606–75240606	1 (0.05%)	1:0	1/0	0.28479919	0.86129537	− 1.106653
15q24 AD	Del	chr15:72670606–75720604	3 (0.15%)	1:2	3/0	0.27613544	0.82152961	− 1.090277
15q24 BD	Del	chr15:73720606–75720604	1 (0.05%)	0:1	1/0	0.30739967	0.68432207	− 0.800280
15q24 BE	Del	chr15:73720606–77840603	2 (0.10%)	0:2	2/0	0.23485125	0.72623183	− 1.128917
15q25.2	Del	chr15:82513967–84070244	5 (0.25%)	0:5	5/0	0.21225081	0.32633295	− 0.430177
16p11.2	Del/Dup	chr16:29641178–30191178	98 (4.96%)	11:87	79/19	0.06570935	1.28740565	− 2.974798
16p11.2 distal	Del/Dup	chr16:28761178–29101178	4 (0.20%)	0:4	3/1	0.12150949	1.61662624	− 2.600350
16p11.2p12.1	Dup	chr16:21341178–29431178	1 (0.05%)	1:0	0/1	0.5534655	2.68382469	− 1.578799
16p13.11	Del/Dup	chr16:15408642–16198642	2 (0.10%)	1:1	1/1	1.67072378	2.46524529	− 0.389038
17p11.2	Del/Dup	chr17:16805961–20576095	71 (3.59%)	44:27	59/12	0.1888066	1.19115966	− 1.841959
17q11.2	Del	chr17:30838856–31888868	62 (3.14%)	10:52	62/0	0.26024285	1.85442774	− 1.963716
17q12	Del	chr17:36460073–37846263	6 (0.30%	4:2	6/0	0.64750654	3.64754421	− 1.728720
17q21.31	Del/Dup	chr17:45626851–46106851	39 (1.97%)	19:20	35/4	0.38234179	0.98304273	− 0.944338
17q23.1q23.2	Del	chr17:59987857–62227857	2 (0.10%)	0:2	2/0	0.56466054	1.30765625	− 0.839748
22q11.2	Del	chr22:18924718–21111383^d^	978 (49.47%)	411:567	978/0	1.45946494	3.69205976	− 0.920692
**All**	**Del/Dup**	**–**	**1977 (100%)**	**870:1107**	**1913/64**	**–**	**–**	**–**

Summarized CNV data. Data are consolidated by locus. BED coordinates correspond to hg38 (LiftOver from hg18 coordinates in Coe et al. [7])

^a^Male to female CNV parent of origin counts

^b^Average male and female recombination rates are the average of the recombination rates calculated for each sample observed for the locus, e.g., 0.123331689 is the average male recombination rate calculated from the male recombination rates of the nine 1q21.1 CNVs

^c^Natural log-transformed average male to female recombination rate ratio for the locus

^d^Breakpoints cited by ClinGen for ~ 3.0 Mb LCR22A-LCR22D interval

### Linear regression analysis

For linear regression, locus-specific estimates for parental origin were derived by combining the data from all published studies for a given locus. To alleviate the uncertainty introduced by small sample sizes, only those loci with more than 10 observations were included. The log_e_-transformed combined male to female parental origin count ratio for each locus was regressed on the calculated average log_e_-transformed average male to female recombination rate ratio for that locus’ CNV interval. Each locus was weighted based on its sample size.

## Results

### Recurrent genomic disorder loci literature search

We conducted a systematic literature search for the 38 non-imprinted and NAHR-mediated CNV loci in Coe et al. [7] (Table 1, Additional file 2: Table S1). We identified parent-of-origin studies that met inclusion criteria as stated in “Methods” section. 77 studies met inclusion criteria; from these 77 studies, data were curated for 24 loci, including copy number variants at 1q21.1 [35–39], 1q21.1 TAR [40], 2q13 [37], 3q29 [37–42], 5q35 [31, 32], 7q11.23 [24, 40, 43–54], 8p23.1 [55, 56], 11q13.2q13.4 [57], 15q13.3 [38, 40, 58], 15q24 (AC, AD, BD, and BE intervals) [59–64], 15q25.2 [65–67],16p11.2 [26, 37, 40, 68–70], distal 16p11.2 [37, 38, 70], 16p11.2p12.1 [71], 16p31.11 [37], 17p11.2 [72–76], 17q11.2 [28, 29, 77], 17q12 [37, 38, 78], 17q21.31 [19, 25, 67, 79–84], 17q23.1q23.2 [69, 85] and 22q11.2 [30, 43, 53, 86–102] (Table 2). Each locus has between one and twenty independent studies representing in total 1977 de novo deletion (N = 1913) and duplication (N = 64) events (Table 2).

### Parent of origin of 3q29 deletion

We determined parent of origin in 12 full trios where a proband had a de novo 3q29 deletion; in 2 additional trios where only proband and maternal DNA samples were available, parent of origin was inferred. For the 12 trios evaluated by SNP arrays, in all cases, the number of Mendelian errors between the presumed inherited (intact) parental allele was zero, and the mean Mendelian errors for the presumed de novo parent of origin allele were 41, with a range of 27–66. For the two trios evaluated with sequence data, Mendelian errors were 20–33-fold elevated when comparing the inherited versus de novo parent. In these 14 trios, 13 deletions (92.9%) arose on the paternal genome indicating a significant departure from the null expectation of 50% (*p* = 0.002, binomial exact). When accounting for only full trios, 11 of 12 (91.7%) deletions arose on paternal haplotypes (*p* = 0.006, binomial exact), altogether indicating there is a paternal bias for origin of the 3q29 deletion (Additional file 1: Table S5). We examined the age distribution of male parents in our cohort; the mean age is 34 years (median = 34 years) and is not significantly different from the 2018 U.S national average, (31.8 years) (*p* = 0.08, Two-tailed two-sample t-test), These data indicate the bias in the 3q29 sample is unlikely to be due to oversampling of older fathers (Additional file 1: Table S5).

### Meiotic recombination and parental origin

**Fig. 1:**
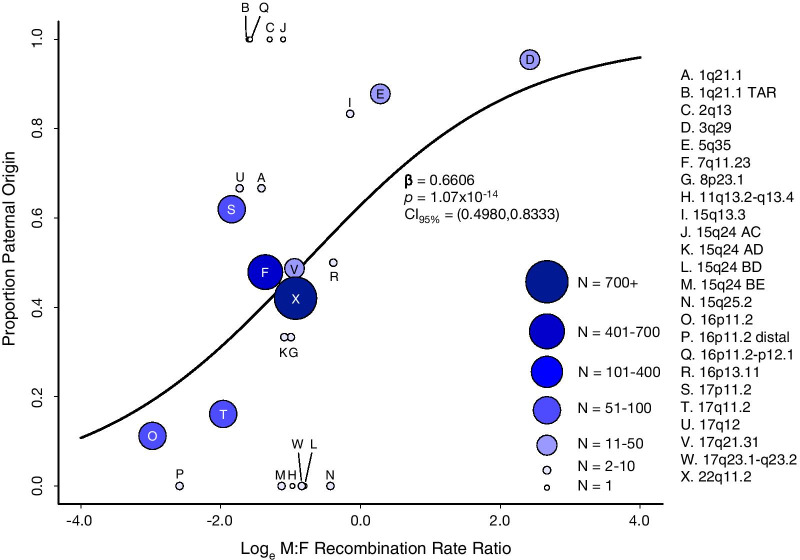
Recombination rates associate with parental origin. Predicted (curve) and observed paternal origin proportions for 1977 CNVs from 24 loci. Curated parent of origin data from 77 published studies are collapsed by loci into single data points; recombination rate ratios are the average of the metric for all CNVs within the data point. Data point size and color correspond to the number of CNV data collapsed into the data point. Recombination rate ratios predict parental origin for CNV mediated by NAHR (*p* = 1.07 × 10^–14^, β = 0.6606, CI_95%_ = (0.4980, 0.8333), OR = 1.936)

We tested the hypothesis that sex-dependent differences in meiotic recombination could explain the parental biases observed for recurrent genomic disorder loci mediated by NAHR. We determined the male and female origin counts of the CNVs curated from the literature search. Of the 1977 CNVs, 870 were paternal in origin and 1107 were of maternal origin. We calculated the average male and female recombination rates (cM/Mb) across the CNV intervals at all 24 loci using recombination rates published by the deCODE genetics group [106] (Additional file 1: Figure S2–S12). We fit a simple logistic model to the data, with the male-to-female recombination rate ratio as the predictor and parental origin as the response variable (Table 2; Additional file 4: Table S4). Our data reveal that the sex-dependent recombination rate ratio significantly predicts parental de novo origin of a given CNV (*p* = 1.07 × 10^–14^, β = 0.6606, CI_95%_ = (0.4980, 0.8333), OR = 1.936) (Fig. 1). In other words: for a given region, the higher the male recombination rate is relative to the female rate, the more likely a CNV formed in that region will be paternal in origin. Stratified analyses on deletions and duplications separately lead to a nearly identical model (Deletions: *p* = 8.88 × 10^–14^, β = 0.6721, CI_95%_ = (0.5009, 0.8546), OR = 1.9584; Duplications: *p* = 0.02, β = 0.8304, CI_95%_ = (0.1508, 1.6017), OR = 2.2942) (Additional file 1: Figure S13–S14, Table S6–S7). Simple linear regression on the subset of CNV loci with more than 10 samples, shows the striking correlation between relative recombination rates and parental origin, where relative recombination rates explain 85% of the variance in parental bias (Additional file 1: Figure S15 and Table S8). Our logistic model can be used to predict paternal origin rates for any locus with estimable recombination in males and females, and we have done so (Additional file 1: Table S9). CNVs at the 15q13.3 and 17q23 both are predicted to have a paternal origin approximately 60% of the time, while at the 16p11.2 distal locus CNVs are predicted to have a maternal origin 76% of the time (Additional file 1: Table S9). If correct, our model would predict these loci exhibit a bias in parental origin.

## Discussion

Parent of origin bias for de novo events at recurrent CNV loci has been well-documented but has lacked a compelling explanation. Our analysis of data gathered on 1977 CNVs from 77 published reports demonstrate that sex-specific variation in local meiotic recombination rates predicts parent of origin at recurrent CNV loci. Human male and female meiotic recombination rates and patterns differ greatly across the broad scale of human chromosomes. Recombination events are nearly uniformly distributed across the chromosome arms in females but tend to be clustered closer to the telomeres in males [108]. We note that this pattern has been previously recognized [26]. Here we have formally tested the hypothesis that recombination variation drives parent of origin variation using a rigorous, statistical framework (Fig. 1) and provided an estimate for the variance in parent of origin bias that is due to sex-specific recombination rates (Additional file 1: Figure S15).

Investigations into the mechanism by which recurrent CNVs arise have focused on LCRs and their makeup [1, 109]. These regions are composed of units of sequence repeats that vary in orientation, percent homology, length, and copy number. Consequently, LCRs are mosaics of varying units, imparting complexity to LCR architecture [23]. The frequency of NAHR events mediated by LCRs is a function of these characteristics and other features of the genomic architecture [21]. Specifically, the rate of NAHR is known to correlate positively with LCR length and percent homology and decrease as the distance between LCRs increases [19, 21]. However, because LCRs are challenging to study with short-read sequencing technology, the population-level variability of these regions is not well described [110]. Recent breakthroughs with long-read sequencing and optical mapping have revealed remarkable variation in LCRs [111–113], and haplotypes with higher risks for CNV formation have now been identified [114]. LCRs are substrates for NAHR [1], and thus are subject to the recombination process. Local recombination rates may influence how likely an NAHR event will happen between two LCRs. Therefore, when analyzing LCR haplotypes and their susceptibility to NAHR, one would need to take into account sex differences in recombination. For example, at loci with maternal biases, specific risk haplotypes may be required for males to form CNVs and vice versa. Greater enrichment of GC content, homologous core duplicons or the PRDM9 motifs, or other recombination-favoring factors may also be required [1, 19].

Variation in recombination rates between sexes is well established [108, 115–118]. Prediction of individual risk may also need to consider individual variation in meiotic recombination, particularly due to heritable variation and the presence or absence of inversion polymorphisms [117, 119]. Variants in several genes, including *PRDM9,* have been shown to affect recombination rates and the distribution of double-stranded breaks in mammals [120, 121]. Common alleles in *PRDM9* are evidenced to affect the percentage of recombination events within individuals that take place at hotspots [120], and variants in *RNF212* are associated with opposite effects on recombination rate between males and females [116, 121]. The unexplained variance in our study may be due to these additional factors, which are rich substrates for future study.

Many human genetic studies have observed correlations between inversion polymorphisms and genomic disorder loci [25, 122]. Because these inversions are copy-number neutral and often located in complex repeat regions, [123] they can be difficult to assay with current high-throughput strategies and their true impact remains to be explored. One model proposes that during meiosis these regions may fail to synapse properly and increase the probability of NAHR [124, 125]. Another theory suggests formation of inversions increases directly oriented content in LCRs leading to an NAHR-favorable haplotype [126]. Supporting these theories, inversion polymorphisms have been identified at the majority of recurrent CNV loci [24, 25, 30, 122, 124, 126, 127]. At the 7q11.23, 17q21.31, and 5q35 loci [24, 25, 127], compelling data indicts inversions as a highly associated marker of CNV formation. However, heterozygous inversions are known to *suppress* recombination perturbing the local pattern of recombination and altering the fate of chiasmata [119]. The analysis presented here strongly suggests that recombination is the driving force for CNV formation giving rise to an alternate explanation for the association between inversions and CNVs; they are both the consequence (and neither one the cause) of recombination between non-allelic homologous LCRs. Inversions and CNVs appear to be associated because both are being initiated by aberrant recombination. Viewing the system in this manner also explains the frequency of individual inversions at CNV loci. Inversions are arising via rare aberrant recombination, like CNVs, but subsequently being driven to higher frequency by natural selection, because they act to suppress recombination and “save offspring” from deleterious genomic disorders. Of course, frequent mutations leading to inversions and the details of LCR structure such as relative orientation and homology within a genomic region may promote or impede CNV formation in a locus-specific manner [128–130]. Further exploration of this relationship with improved genomic mapping can test these alternative models [131]. One testable prediction of the model described here is that inversions should be at higher frequency at loci giving rise to highly deleterious CNVs, as opposed to loci harboring recurrent benign CNVs.

To our knowledge, this study is the first comprehensive investigation of parental origin of recurrent, NAHR-mediated CNV loci. Investigations of predominantly *nonrecurrent* CNVs show paternal bias [132–134]. Unlike recurrent CNVs, nonrecurrent CNVs are mostly formed via non-homologous end joining (NHEJ) and replicative mechanisms [1, 135, 136]. The standing hypothesis is that replication-based mechanisms of nonrecurrent CNV formation, which are known to accumulate errors in male germlines, contribute to this bias [132]. Our study reinforces the idea that the factors influencing recurrent CNVs differ from those impacting nonrecurrent CNVs. Future genome-wide analyses with larger sample sizes can further help refine our understanding of the divergent forces at play affecting recurrent and nonrecurrent CNV formation.

We conducted a comprehensive literature search at 38 loci and ultimately identified 1977 samples for analysis. We note that the majority of the data come from 7 well-studied loci (Table 1). While we thoroughly curated the data in a systematic way, it is possible that our data is subject to publication bias, where loci that exhibit parent of origin biases are more likely to have parental origin reported. Further exacerbating potential publication bias, genetic testing for the affected patient (and even more so for the parents) can be difficult to obtain due to concerns such as insurance coverage, potential future discrimination, and privacy concerns [137–140]. However, we note individuals with CNVs are generally not ascertained or recruited under the expectation that recombination affects parent of origin, and therefore, any potential publication or ascertainment bias is unlikely to confound the results of our analysis. Analysis of a larger cohort of CNV loci including benign CNVs will give greater insight into the role of recombination, and sex differences in recombination influencing parent of origin in CNVs.

Our estimates of recombination rates summarize CNV-scale (broad-scale) patterns of recombination, rather than fine-scale patterns near the sites of relevant recombination events that form these CNVs—LCRs. For example, local sex-specific hotspots within LCRs could be the underlying drivers behind the correlation between recombination rates and parental origin. Given the nature of repetitive regions like LCRs and our inability to adequately interrogate them with current sequencing technologies, accurate recombination data across and within the LCR regions is not available. In other words, the data is currently insufficient to conclude whether or not these broad-scale patterns are tightly correlated with fine-scale recombination rates in and around the LCRs. The best available data in the field allows us to infer the following: broad-scale patterns of recombination tightly predict patterns of parental origin.

## Conclusions

In this study, we determined male and female differences in meiotic recombination rates significantly predict parent of origin for recurrent CNV loci. Combining the sex-specific recombination landscape and the mechanistic factors underlying it with a more detailed understanding of existing structural factors at genomic disorder loci can be expected to help guide standards used to identify and perform genetic counseling for individuals at risk of genomic rearrangement.

## Supplementary Information

**Additional file 1: Figure S1.** Schematic of recombination rate calculation method; **Figures S2–S12.** Recombination rates of 24 loci analyzed; **Figure S13.** Logistic regression with deletions only; **Figure S14.** Logistic regression with duplications only; **Figure S15.** Linear regression with combined CNV parent of origin data; **Table S3.** LCR22 recombination rate data; **Table S5.** Demographic data for 3q29 cohort; **Table S6.** Summarized data for logistic regression with deletions only; **Table S7.** Summarized data for logistic regression with duplications only; **Table S8.** Sensitivity analysis results for linear regression analysis with deletions and duplications combined; **Table S9.** Predicted paternal origin probability for loci with small sample sizes or missing from analysis; Supplemental Materials and Methods; Supplemental References.

**Additional file 2: Table S1.** Exclusion/Inclusion statuses and literature search results of genomic disorder loci conducted January 2021.

**Additional file 3: Table S2.** List of 1268 search results curated from literature search.

**Additional file 4: Table S4.** Logistic regression data for 1977 CNVs.

## Data Availability

The majority of the parent of origin data in our analyses was extracted from published sources, as in Table 1. The recombination rate datasets analyzed during the current study are available at https://doi.org/10.1126/science.aau1043 [106]. The SNP data generated and analyzed at the 3q29 locus is available in the NIMH Data Archive repository, (https://nda.nih.gov), collection ID #2614. The data will also be provided upon request to qualified investigators; please email the corresponding author (jmulle@emory.edu) for data access.

## Abbreviations

CNV: Copy number variant
LCR: Low-copy repeat
NAHR: Non-allelic homologous recombination
NCHS: National Center for Health Statistics

## References

[1] Carvalho CM, Lupski JR (2016). Mechanisms underlying structural variant formation in genomic disorders. Nat Rev Genet.

[2] Girirajan S, Rosenfeld JA, Coe BP, Parikh S, Friedman N, Goldstein A (2012). Phenotypic heterogeneity of genomic disorders and rare copy-number variants. N Engl J Med.

[3] Harel T, Lupski JR (2018). Genomic disorders 20 years on-mechanisms for clinical manifestations. Clin Genet.

[4] Mefford HC (2009). Genotype to phenotype-discovery and characterization of novel genomic disorders in a “genotype-first” era. Genet Med.

[5] Feuk L, Carson AR, Scherer SW (2006). Structural variation in the human genome. Nat Rev Genet.

[6] Girirajan S, Campbell CD, Eichler EE (2011). Human copy number variation and complex genetic disease. Annu Rev Genet.

[7] Coe BP, Witherspoon K, Rosenfeld JA, van Bon BW, Vulto-van Silfhout AT, Bosco P (2014). Refining analyses of copy number variation identifies specific genes associated with developmental delay. Nat Genet.

[8] Kaminsky EB, Kaul V, Paschall J, Church DM, Bunke B, Kunig D (2011). An evidence-based approach to establish the functional and clinical significance of copy number variants in intellectual and developmental disabilities. Genet Med.

[9] Lifton RP, Dluhy RG, Powers M, Rich GM, Cook S, Ulick S (1992). A chimaeric 11 beta-hydroxylase/aldosterone synthase gene causes glucocorticoid-remediable aldosteronism and human hypertension. Nature.

[10] Mulle JG, Dodd AF, McGrath JA, Wolyniec PS, Mitchell AA, Shetty AC (2010). Microdeletions of 3q29 confer high risk for schizophrenia. Am J Hum Genet.

[11] Rovelet-Lecrux A, Hannequin D, Raux G, Le Meur N, Laquerriere A, Vital A (2006). APP locus duplication causes autosomal dominant early-onset Alzheimer disease with cerebral amyloid angiopathy. Nat Genet.

[12] Walters RG, Jacquemont S, Valsesia A, de Smith AJ, Martinet D, Andersson J (2010). A new highly penetrant form of obesity due to deletions on chromosome 16p11.2. Nature.

[13] Pinto D, Delaby E, Merico D, Barbosa M, Merikangas A, Klei L (2014). Convergence of genes and cellular pathways dysregulated in autism spectrum disorders. Am J Hum Genet.

[14] Sebat J, Lakshmi B, Malhotra D, Troge J, Lese-Martin C, Walsh T (2007). Strong association of de novo copy number mutations with autism. Science.

[15] Marshall CR, Howrigan DP, Merico D, Thiruvahindrapuram B, Wu W, Greer DS (2017). Contribution of copy number variants to schizophrenia from a genome-wide study of 41,321 subjects. Nat Genet.

[16] Walsh T, McClellan JM, McCarthy SE, Addington AM, Pierce SB, Cooper GM (2008). Rare structural variants disrupt multiple genes in neurodevelopmental pathways in schizophrenia. Science.

[17] Girirajan S, Brkanac Z, Coe BP, Baker C, Vives L, Vu TH (2011). Relative burden of large CNVs on a range of neurodevelopmental phenotypes. PLoS Genet.

[18] Dittwald P, Gambin T, Szafranski P, Li J, Amato S, Divon MY (2013). NAHR-mediated copy-number variants in a clinical population: mechanistic insights into both genomic disorders and Mendelizing traits. Genome Res.

[19] Sharp AJ, Hansen S, Selzer RR, Cheng Z, Regan R, Hurst JA (2006). Discovery of previously unidentified genomic disorders from the duplication architecture of the human genome. Nat Genet.

[20] Liu P, Carvalho CM, Hastings PJ, Lupski JR (2012). Mechanisms for recurrent and complex human genomic rearrangements. Curr Opin Genet Dev.

[21] Liu P, Lacaria M, Zhang F, Withers M, Hastings PJ, Lupski JR (2011). Frequency of nonallelic homologous recombination is correlated with length of homology: evidence that ectopic synapsis precedes ectopic crossing-over. Am J Hum Genet.

[22] Stankiewicz P, Lupski JR (2010). Structural variation in the human genome and its role in disease. Annu Rev Med.

[23] Marques-Bonet T, Eichler EE (2009). The evolution of human segmental duplications and the core duplicon hypothesis. Cold Spring Harb Symp Quant Biol.

[24] Hobart HH, Morris CA, Mervis CB, Pani AM, Kistler DJ, Rios CM (2010). Inversion of the Williams syndrome region is a common polymorphism found more frequently in parents of children with Williams syndrome. Am J Med Genet C Semin Med Genet.

[25] Koolen DA, Vissers LE, Pfundt R, de Leeuw N, Knight SJ, Regan R (2006). A new chromosome 17q21.31 microdeletion syndrome associated with a common inversion polymorphism. Nat Genet.

[26] Duyzend MH, Nuttle X, Coe BP, Baker C, Nickerson DA, Bernier R (2016). Maternal modifiers and parent-of-origin bias of the autism-associated 16p11.2 CNV. Am J Hum Genet.

[27] Lazaro C, Gaona A, Ainsworth P, Tenconi R, Vidaud D, Kruyer H (1996). Sex differences in mutational rate and mutational mechanism in the NF1 gene in neurofibromatosis type 1 patients. Hum Genet.

[28] Neuhausler L, Summerer A, Cooper DN, Mautner VF, Kehrer-Sawatzki H (2018). Pronounced maternal parent-of-origin bias for type-1 NF1 microdeletions. Hum Genet.

[29] Upadhyaya M, Ruggieri M, Maynard J, Osborn M, Hartog C, Mudd S (1998). Gross deletions of the neurofibromatosis type 1 (NF1) gene are predominantly of maternal origin and commonly associated with a learning disability, dysmorphic features and developmental delay. Hum Genet.

[30] Delio M, Guo T, McDonald-McGinn DM, Zackai E, Herman S, Kaminetzky M (2013). Enhanced maternal origin of the 22q11.2 deletion in velocardiofacial and DiGeorge syndromes. Am J Hum Genet.

[31] Miyake N, Kurotaki N, Sugawara H, Shimokawa O, Harada N, Kondoh T (2003). Preferential paternal origin of microdeletions caused by prezygotic chromosome or chromatid rearrangements in Sotos syndrome. Am J Hum Genet.

[32] Tatton-Brown K, Douglas J, Coleman K, Baujat G, Chandler K, Clarke A (2005). Multiple mechanisms are implicated in the generation of 5q35 microdeletions in Sotos syndrome. J Med Genet.

[33] Page SL, Hawley RS (2003). Chromosome choreography: the meiotic ballet. Science.

[34] Hunt PA, Hassold TJ (2002). Sex matters in meiosis. Science.

[35] Christiansen J, Dyck JD, Elyas BG, Lilley M, Bamforth JS, Hicks M (2004). Chromosome 1q21.1 contiguous gene deletion is associated with congenital heart disease. Circ Res.

[36] Mefford HC, Sharp AJ, Baker C, Itsara A, Jiang Z, Buysse K (2008). Recurrent rearrangements of chromosome 1q21.1 and variable pediatric phenotypes. N Engl J Med.

[37] Sanders SJ, He X, Willsey AJ, Ercan-Sencicek AG, Samocha KE, Cicek AE (2015). Insights into autism spectrum disorder genomic architecture and biology from 71 risk loci. Neuron.

[38] Smajlagic D, Lavrichenko K, Berland S, Helgeland O, Knudsen GP, Vaudel M (2021). Population prevalence and inheritance pattern of recurrent CNVs associated with neurodevelopmental disorders in 12,252 newborns and their parents. Eur J Hum Genet.

[39] Soemedi R, Wilson IJ, Bentham J, Darlay R, Topf A, Zelenika D (2012). Contribution of global rare copy-number variants to the risk of sporadic congenital heart disease. Am J Hum Genet.

[40] Kirov G, Pocklington AJ, Holmans P, Ivanov D, Ikeda M, Ruderfer D (2012). De novo CNV analysis implicates specific abnormalities of postsynaptic signalling complexes in the pathogenesis of schizophrenia. Mol Psychiatry.

[41] Malt EA, Juhasz K, Frengen A, Wangensteen T, Emilsen NM, Hansen B (2019). Neuropsychiatric phenotype in relation to gene variants in the hemizygous allele in 3q29 deletion carriers: a case series. Mol Genet Genomic Med.

[42] Quintero-Rivera F, Sharifi-Hannauer P, Martinez-Agosto JA (2010). Autistic and psychiatric findings associated with the 3q29 microdeletion syndrome: case report and review. Am J Med Genet A.

[43] Baumer A, Dutly F, Balmer D, Riegel M, Tukel T, Krajewska-Walasek M (1998). High level of unequal meiotic crossovers at the origin of the 22q11.2 and 7q11.23 deletions. Hum Mol Genet.

[44] Bayes M, Magano LF, Rivera N, Flores R, Perez Jurado LA (2003). Mutational mechanisms of Williams–Beuren syndrome deletions. Am J Hum Genet.

[45] Codina-Sola M, Costa-Roger M, Perez-Garcia D, Flores R, Palacios-Verdu MG, Cusco I (2019). Genetic factors contributing to autism spectrum disorder in Williams–Beuren syndrome. J Med Genet.

[46] Depienne C, Heron D, Betancur C, Benyahia B, Trouillard O, Bouteiller D (2007). Autism, language delay and mental retardation in a patient with 7q11 duplication. J Med Genet.

[47] Dutra RL, Pieri Pde C, Teixeira AC, Honjo RS, Bertola DR, Kim CA (2011). Detection of deletions at 7q11.23 in Williams–Beuren syndrome by polymorphic markers. Clinics (Sao Paulo).

[48] Ghaffari M, Tahmasebi Birgani M, Kariminejad R, Saberi A (2018). Genotype–phenotype correlation and the size of microdeletion or microduplication of 7q11.23 region in patients with Williams–Beuren syndrome. Ann Hum Genet.

[49] Masson J, Demily C, Chatron N, Labalme A, Rollat-Farnier PA, Schluth-Bolard C (2019). Molecular investigation, using chromosomal microarray and whole exome sequencing, of six patients affected by Williams Beuren syndrome and Autism Spectrum Disorder. Orphanet J Rare Dis.

[50] Morris CA, Mervis CB, Paciorkowski AP, Abdul-Rahman O, Dugan SL, Rope AF (2015). 7q11.23 duplication syndrome: physical characteristics and natural history. Am J Med Genet A.

[51] Perez-Garcia D, Flores R, Brun-Gasca C, Perez-Jurado LA (2015). Lateral preference in Williams–Beuren syndrome is associated with cognition and language. Eur Child Adolesc Psychiatry.

[52] Robinson WP, Waslynka J, Bernasconi F, Wang M, Clark S, Kotzot D (1996). Delineation of 7q11.2 deletions associated with Williams–Beuren syndrome and mapping of a repetitive sequence to within and to either side of the common deletion. Genomics.

[53] Thomas NS, Durkie M, Potts G, Sandford R, Van Zyl B, Youings S (2006). Parental and chromosomal origins of microdeletion and duplication syndromes involving 7q11.23, 15q11-q13 and 22q11. Eur J Hum Genet.

[54] Wu YQ, Sutton VR, Nickerson E, Lupski JR, Potocki L, Korenberg JR (1998). Delineation of the common critical region in Williams syndrome and clinical correlation of growth, heart defects, ethnicity, and parental origin. Am J Med Genet.

[55] Barber JC, Rosenfeld JA, Foulds N, Laird S, Bateman MS, Thomas NS (2013). 8p23.1 duplication syndrome; common, confirmed, and novel features in six further patients. Am J Med Genet A.

[56] Shimokawa O, Miyake N, Yoshimura T, Sosonkina N, Harada N, Mizuguchi T (2005). Molecular characterization of del(8)(p23.1p23.1) in a case of congenital diaphragmatic hernia. Am J Med Genet A.

[57] Wischmeijer A, Magini P, Giorda R, Gnoli M, Ciccone R, Cecconi L (2011). Olfactory receptor-related duplicons mediate a microdeletion at 11q13.2q13.4 associated with a syndromic phenotype. Mol Syndromol.

[58] Sharp AJ, Mefford HC, Li K, Baker C, Skinner C, Stevenson RE (2008). A recurrent 15q13.3 microdeletion syndrome associated with mental retardation and seizures. Nat Genet.

[59] Chen CP, Wang LK, Chern SR, Wu PS, Chen SW, Wu FT (2020). Prenatal diagnosis and molecular cytogenetic characterization of a chromosome 15q24 microdeletion. Taiwan J Obstet Gynecol.

[60] Gao X, Gotway G, Rathjen K, Johnston C, Sparagana S, Wise CA (2014). Genomic analyses of patients with unexplained early-onset scoliosis. Spine Deform.

[61] Huynh MT, Lambert AS, Tosca L, Petit F, Philippe C, Parisot F (2018). 15q24.1 BP4–BP1 microdeletion unmasking paternally inherited functional polymorphisms combined with distal 15q24.2q24.3 duplication in a patient with epilepsy, psychomotor delay, overweight, ventricular arrhythmia. Eur J Med Genet.

[62] McInnes LA, Nakamine A, Pilorge M, Brandt T, Jimenez Gonzalez P, Fallas M (2010). A large-scale survey of the novel 15q24 microdeletion syndrome in autism spectrum disorders identifies an atypical deletion that narrows the critical region. Mol Autism.

[63] Mefford HC, Rosenfeld JA, Shur N, Slavotinek AM, Cox VA, Hennekam RC (2012). Further clinical and molecular delineation of the 15q24 microdeletion syndrome. J Med Genet.

[64] Sharp AJ, Selzer RR, Veltman JA, Gimelli S, Gimelli G, Striano P (2007). Characterization of a recurrent 15q24 microdeletion syndrome. Hum Mol Genet.

[65] Burgess T, Brown NJ, Stark Z, Bruno DL, Oertel R, Chong B (2014). Characterization of core clinical phenotypes associated with recurrent proximal 15q25.2 microdeletions. Am J Med Genet A.

[66] Palumbo O, Palumbo P, Palladino T, Stallone R, Miroballo M, Piemontese MR (2012). An emerging phenotype of interstitial 15q25.2 microdeletions: clinical report and review. Am J Med Genet A.

[67] Wagenstaller J, Spranger S, Lorenz-Depiereux B, Kazmierczak B, Nathrath M, Wahl D (2007). Copy-number variations measured by single-nucleotide-polymorphism oligonucleotide arrays in patients with mental retardation. Am J Hum Genet.

[68] Egolf LE, Vaksman Z, Lopez G, Rokita JL, Modi A, Basta PV (2019). Germline 16p11.2 microdeletion predisposes to neuroblastoma. Am J Hum Genet.

[69] Karolak JA, Gambin T, Honey EM, Slavik T, Popek E, Stankiewicz P (2020). A de novo 2.2 Mb recurrent 17q23.1q23.2 deletion unmasks novel putative regulatory non-coding SNVs associated with lethal lung hypoplasia and pulmonary hypertension: a case report. BMC Med Genomics.

[70] Redaelli S, Maitz S, Crosti F, Sala E, Villa N, Spaccini L (2019). Refining the phenotype of recurrent rearrangements of chromosome 16. Int J Mol Sci.

[71] Tabet AC, Pilorge M, Delorme R, Amsellem F, Pinard JM, Leboyer M (2012). Autism multiplex family with 16p11.2p12.2 microduplication syndrome in monozygotic twins and distal 16p11.2 deletion in their brother. Eur J Hum Genet.

[72] Greenberg F, Guzzetta V, Montes de Oca-Luna R, Magenis RE, Smith AC, Richter SF (1991). Molecular analysis of the Smith–Magenis syndrome: a possible contiguous-gene syndrome associated with del(17)(p11.2). Am J Hum Genet.

[73] Nakamine A, Ouchanov L, Jimenez P, Manghi ER, Esquivel M, Monge S (2008). Duplication of 17(p11.2p11.2) in a male child with autism and severe language delay. Am J Med Genet A.

[74] Potocki L, Shaw CJ, Stankiewicz P, Lupski JR (2003). Variability in clinical phenotype despite common chromosomal deletion in Smith–Magenis syndrome [del(17)(p11.2p11.2)]. Genet Med.

[75] Shaw CJ, Bi W, Lupski JR (2002). Genetic proof of unequal meiotic crossovers in reciprocal deletion and duplication of 17p11.2. Am J Hum Genet.

[76] Yang SP, Bidichandani SI, Figuera LE, Juyal RC, Saxon PJ, Baldini A (1997). Molecular analysis of deletion (17)(p11.2p11.2) in a family segregating a 17p paracentric inversion: implications for carriers of paracentric inversions. Am J Hum Genet.

[77] Steinmann K, Kluwe L, Cooper DN, Brems H, De Raedt T, Legius E (2008). Copy number variations in the NF1 gene region are infrequent and do not predispose to recurrent type-1 deletions. Eur J Hum Genet.

[78] Palumbo P, Antona V, Palumbo O, Piccione M, Nardello R, Fontana A (2014). Variable phenotype in 17q12 microdeletions: clinical and molecular characterization of a new case. Gene.

[79] Digilio MC, Bernardini L, Capolino R, Digilio M, Dentici ML, Novelli A (2014). Hypopigmented skin patches in 17q21.31 microdeletion syndrome: expanding the spectrum of cutaneous findings. Clin Dysmorphol.

[80] Dubourg C, Sanlaville D, Doco-Fenzy M, Le Caignec C, Missirian C, Jaillard S (2011). Clinical and molecular characterization of 17q21.31 microdeletion syndrome in 14 French patients with mental retardation. Eur J Med Genet.

[81] Grisart B, Willatt L, Destree A, Fryns JP, Rack K, de Ravel T (2009). 17q21.31 microduplication patients are characterised by behavioural problems and poor social interaction. J Med Genet.

[82] Kirchhoff M, Bisgaard AM, Duno M, Hansen FJ, Schwartz M (2007). A 17q21.31 microduplication, reciprocal to the newly described 17q21.31 microdeletion, in a girl with severe psychomotor developmental delay and dysmorphic craniofacial features. Eur J Med Genet.

[83] Koolen DA, Sharp AJ, Hurst JA, Firth HV, Knight SJ, Goldenberg A (2008). Clinical and molecular delineation of the 17q21.31 microdeletion syndrome. J Med Genet.

[84] Vlckova M, Hancarova M, Drabova J, Slamova Z, Koudova M, Alanova R (2014). Monozygotic twins with 17q21.31 microdeletion syndrome. Twin Res Hum Genet.

[85] Karolak JA, Vincent M, Deutsch G, Gambin T, Cogne B, Pichon O (2019). Complex compound inheritance of lethal lung developmental disorders due to disruption of the TBX-FGF pathway. Am J Hum Genet.

[86] Bassett AS, Marshall CR, Lionel AC, Chow EW, Scherer SW (2008). Copy number variations and risk for schizophrenia in 22q11.2 deletion syndrome. Hum Mol Genet.

[87] Baumer A, Riegel M, Schinzel A (2004). Non-random asynchronous replication at 22q11.2 favours unequal meiotic crossovers leading to the human 22q11.2 deletion. J Med Genet.

[88] Brondum-Nielsen K, Christensen K (1996). Chromosome 22q11 deletion and other chromosome aberrations in cases with cleft palate, congenital heart defects and/or mental disability. A survey based on the Danish Facial Cleft Register. Clin Genet.

[89] Chakraborty D, Bernal AJ, Schoch K, Howard TD, Ip EH, Hooper SR (2012). Dysregulation of DGCR6 and DGCR6L: psychopathological outcomes in chromosome 22q11.2 deletion syndrome. Transl Psychiatry.

[90] Demczuk S, Levy A, Aubry M, Croquette MF, Philip N, Prieur M (1995). Excess of deletions of maternal origin in the DiGeorge/velo-cardio-facial syndromes. A study of 22 new patients and review of the literature. Hum Genet.

[91] Fokstuen S, Arbenz U, Artan S, Dutly F, Bauersfeld U, Brecevic L (1998). 22q11.2 deletions in a series of patients with non-selective congenital heart defects: incidence, type of defects and parental origin. Clin Genet.

[92] Glaser B, Mumme DL, Blasey C, Morris MA, Dahoun SP, Antonarakis SE (2002). Language skills in children with velocardiofacial syndrome (deletion 22q11.2). J Pediatr.

[93] Guo T, Diacou A, Nomaru H, McDonald-McGinn DM, Hestand M, Demaerel W (2018). Deletion size analysis of 1680 22q11.2DS subjects identifies a new recombination hotspot on chromosome 22q11.2. Hum Mol Genet.

[94] Michaelovsky E, Gothelf D, Korostishevsky M, Frisch A, Burg M, Carmel M (2008). Association between a common haplotype in the COMT gene region and psychiatric disorders in individuals with 22q11.2DS. Int J Neuropsychopharmacol.

[95] Molck MC, Vieira TP, Simioni M, Sgardioli IC, dos Santos AP, Xavier AC (2015). Distal 22q11.2 microduplication combined with typical 22q11.2 proximal deletion: a case report. Am J Med Genet A.

[96] Morrow B, Goldberg R, Carlson C, Das Gupta R, Sirotkin H, Collins J (1995). Molecular definition of the 22q11 deletions in velo-cardio-facial syndrome. Am J Hum Genet.

[97] Saitta SC, Harris SE, Gaeth AP, Driscoll DA, McDonald-McGinn DM, Maisenbacher MK (2004). Aberrant interchromosomal exchanges are the predominant cause of the 22q11.2 deletion. Hum Mol Genet.

[98] Saitta SC, Harris SE, McDonald-McGinn DM, Emanuel BS, Tonnesen MK, Zackai EH (2004). Independent de novo 22q11.2 deletions in first cousins with DiGeorge/velocardiofacial syndrome. Am J Med Genet A.

[99] Sandrin-Garcia P, Abramides DV, Martelli LR, Ramos ES, Richieri-Costa A, Passos GA (2007). Typical phenotypic spectrum of velocardiofacial syndrome occurs independently of deletion size in chromosome 22q11.2. Mol Cell Biochem.

[100] Sandrin-Garcia P, Macedo C, Martelli LR, Ramos ES, Guion-Almeida ML, Richieri-Costa A (2002). Recurrent 22q11.2 deletion in a sibship suggestive of parental germline mosaicism in velocardiofacial syndrome. Clin Genet.

[101] Vervoort L, Demaerel W, Rengifo LY, Odrzywolski A, Vergaelen E, Hestand MS (2019). Atypical chromosome 22q11.2 deletions are complex rearrangements and have different mechanistic origins. Hum Mol Genet.

[102] Vittorini S, Sacchelli M, Iascone MR, Collavoli A, Storti S, Giusti A (2001). Molecular characterization of chromosome 22 deletions by short tandem repeat polymorphism (STRP) in patients with conotruncal heart defects. Clin Chem Lab Med.

[103] Murphy MM, Lindsey Burrell T, Cubells JF, Espana RA, Gambello MJ, Goines KCB (2018). Study protocol for The Emory 3q29 Project: evaluation of neurodevelopmental, psychiatric, and medical symptoms in 3q29 deletion syndrome. BMC Psychiatry.

[104] Chang CC, Chow CC, Tellier LC, Vattikuti S, Purcell SM, Lee JJ (2015). Second-generation PLINK: rising to the challenge of larger and richer datasets. Gigascience.

[105] Team RC (2014). R: a language and environment for statistical computing.

[106] Halldorsson BV, Palsson G, Stefansson OA, Jonsson H, Hardarson MT, Eggertsson HP (2019). Characterizing mutagenic effects of recombination through a sequence-level genetic map. Science.

[107] McDonald-McGinn DM, Hain HS, Emanuel BS, Zackai EH, Adam MP, Ardinger HH, Pagon RA, Wallace SE, Bean LJH, Mirzaa G (1993). 22q11.2 deletion syndrome. GeneReviews((R)).

[108] Broman KW, Murray JC, Sheffield VC, White RL, Weber JL (1998). Comprehensive human genetic maps: individual and sex-specific variation in recombination. Am J Hum Genet.

[109] Sharp AJ, Cheng Z, Eichler EE (2006). Structural variation of the human genome. Nature Rev. Genet..

[110] Eichler EE (1998). Masquerading repeats: paralogous pitfalls of the human genome. Genome Res.

[111] Lam ET, Hastie A, Lin C, Ehrlich D, Das SK, Austin MD (2012). Genome mapping on nanochannel arrays for structural variation analysis and sequence assembly. Nat Biotechnol.

[112] Levy-Sakin M, Pastor S, Mostovoy Y, Li L, Leung AKY, McCaffrey J (2019). Genome maps across 26 human populations reveal population-specific patterns of structural variation. Nat Commun.

[113] Mak AC, Lai YY, Lam ET, Kwok TP, Leung AK, Poon A (2016). Genome-wide structural variation detection by genome mapping on nanochannel arrays. Genetics.

[114] Demaerel W, Mostovoy Y, Yilmaz F, Vervoort L, Pastor S, Hestand MS (2019). The 22q11 low copy repeats are characterized by unprecedented size and structural variability. Genome Res.

[115] Agarwal I, Przeworski M (2019). Signatures of replication timing, recombination, and sex in the spectrum of rare variants on the human X chromosome and autosomes. Proc Natl Acad Sci U S A.

[116] Chowdhury R, Bois PR, Feingold E, Sherman SL, Cheung VG (2009). Genetic analysis of variation in human meiotic recombination. PLoS Genet.

[117] Coop G, Wen X, Ober C, Pritchard JK, Przeworski M (2008). High-resolution mapping of crossovers reveals extensive variation in fine-scale recombination patterns among humans. Science.

[118] Kong A, Gudbjartsson DF, Sainz J, Jonsdottir GM, Gudjonsson SA, Richardsson B (2002). A high-resolution recombination map of the human genome. Nat Genet.

[119] Crown KN, Miller DE, Sekelsky J, Hawley RS (2018). Local inversion heterozygosity alters recombination throughout the genome. Curr Biol.

[120] Kong A, Thorleifsson G, Gudbjartsson DF, Masson G, Sigurdsson A, Jonasdottir A (2010). Fine-scale recombination rate differences between sexes, populations and individuals. Nature.

[121] Kong A, Thorleifsson G, Stefansson H, Masson G, Helgason A, Gudbjartsson DF (2008). Sequence variants in the RNF212 gene associate with genome-wide recombination rate. Science.

[122] Antonacci F, Kidd JM, Marques-Bonet T, Ventura M, Siswara P, Jiang Z (2009). Characterization of six human disease-associated inversion polymorphisms. Hum Mol Genet.

[123] Puig M, Casillas S, Villatoro S, Caceres M (2015). Human inversions and their functional consequences. Brief Funct Genomics.

[124] Osborne LR, Li M, Pober B, Chitayat D, Bodurtha J, Mandel A (2001). A 1.5 million-base pair inversion polymorphism in families with Williams–Beuren syndrome. Nat Genet.

[125] Rao PN, Li W, Vissers LE, Veltman JA, Ophoff RA (2010). Recurrent inversion events at 17q21.31 microdeletion locus are linked to the MAPT H2 haplotype. Cytogenet Genome Res.

[126] Gimelli G, Pujana MA, Patricelli MG, Russo S, Giardino D, Larizza L (2003). Genomic inversions of human chromosome 15q11-q13 in mothers of Angelman syndrome patients with class II (BP2/3) deletions. Hum Mol Genet.

[127] Visser R, Shimokawa O, Harada N, Kinoshita A, Ohta T, Niikawa N (2005). Identification of a 3.0-kb major recombination hotspot in patients with Sotos syndrome who carry a common 1.9-Mb microdeletion. Am J Hum Genet.

[128] Antonarakis SE, Rossiter JP, Young M, Horst J, de Moerloose P, Sommer SS (1995). Factor VIII gene inversions in severe hemophilia A: results of an international consortium study. Blood.

[129] Tam E, Young EJ, Morris CA, Marshall CR, Loo W, Scherer SW (2008). The common inversion of the Williams–Beuren syndrome region at 7q11.23 does not cause clinical symptoms. Am J Med Genet A.

[130] Zody MC, Jiang Z, Fung HC, Antonacci F, Hillier LW, Cardone MF (2008). Evolutionary toggling of the MAPT 17q21.31 inversion region. Nat Genet.

[131] Giner-Delgado C, Villatoro S, Lerga-Jaso J, Gaya-Vidal M, Oliva M, Castellano D (2019). Evolutionary and functional impact of common polymorphic inversions in the human genome. Nat Commun.

[132] Hehir-Kwa JY, Rodriguez-Santiago B, Vissers LE, de Leeuw N, Pfundt R, Buitelaar JK (2011). De novo copy number variants associated with intellectual disability have a paternal origin and age bias. J Med Genet.

[133] Kloosterman WP, Francioli LC, Hormozdiari F, Marschall T, Hehir-Kwa JY, Abdellaoui A (2015). Characteristics of de novo structural changes in the human genome. Genome Res.

[134] Ma R, Deng L, Xia Y, Wei X, Cao Y, Guo R (2017). A clear bias in parental origin of de novo pathogenic CNVs related to intellectual disability, developmental delay and multiple congenital anomalies. Sci Rep.

[135] Lee JA, Carvalho CM, Lupski JR (2007). A DNA replication mechanism for generating nonrecurrent rearrangements associated with genomic disorders. Cell.

[136] Zhang F, Khajavi M, Connolly AM, Towne CF, Batish SD, Lupski JR (2009). The DNA replication FoSTeS/MMBIR mechanism can generate genomic, genic and exonic complex rearrangements in humans. Nat Genet.

[137] Barton KS, Tabor HK, Starks H, Garrison NA, Laurino M, Burke W (2018). Pathways from autism spectrum disorder diagnosis to genetic testing. Genet Med.

[138] Glassford MR, Rosenfeld JA, Freedman AA, Zwick ME, Mulle JG, Unique Rare Chromosome Disorder Support G (2016). Novel features of 3q29 deletion syndrome: results from the 3q29 registry. Am J Med Genet A.

[139] Phillips KA, Trosman JR, Deverka PA, Quinn B, Tunis S, Neumann PJ (2018). Insurance coverage for genomic tests. Science.

[140] Stiles D, Appelbaum PS (2019). Cases in precision medicine: concerns about privacy and discrimination after genomic sequencing. Ann Intern Med.

