# Supplementary material for: Sex-specific recombination patterns predict parent of origin for recurrent genomic disorders
Source: BMC Med Genomics. 2021 Jun 9;14:154. doi: 10.1186/s12920-021-00999-8 (PMC8190997; doi:10.1186/s12920-021-00999-8)
Supplement: Supplementary file 1 — Additional file 1: Figure S1. Schematic of recombination rate calculation method; Figures S2–S12. Recombination rates of 24 loci analyzed; Figure S13. Logistic regression with deletions only; Figure S14. Logistic regression with duplications only; Figure S15. Linear regression with combined CNV parent of origin data; Table S3. LCR22 recombination rate data; Table S5. Demographic data for 3q29 cohort; Table S6. Summarized data for logistic regression with deletions only; Table S7. Summarized data for logistic regression with duplications only; Table S8. Sensitivity analysis results for linear regression analysis with deletions and duplications combined; Table S9. Predicted paternal origin probability for loci with small sample sizes or missing from analysis; Supplemental Materials and Methods; Supplemental References. [file 12920_2021_999_MOESM1_ESM.docx]

**SUPPLEMENTAL DATA**

**Supplemental Figures**

S1. Schematic of recombination rate calculations

S2. Recombination across 1q21.1 TAR and 1q21.1 regions

S3. Recombination across 2q13 and 3q29 regions

S4. Recombination across 5q35 and 7q11.23 regions

S5. Recombination across 8p23.1 and 11q13.2q13.4 regions

S6. Recombination across 15q13.3 and 15q24 regions

S7. Recombination across 15q25.2 and 16p11.2 regions

S8. Recombination across 16p11.2 distal and 16p11.2p12.1 regions

S9. Recombination across 16p13.11 and 17p11.2 regions

S10. Recombination across 17q11.2 and17q12 regions

S11. Recombination across 17q21.31 and 17q23.1q23.2 regions

S12. Recombination across 22q11.2 region

S13. Logistic regression with deletions only

S14. Logistic regression with duplications only

S15. Linear regression with combined CNV parent of origin data

**Supplemental Tables**

S1. Exclusion/Inclusion statuses and literature search results of genomic disorder loci conducted January 2021 (Excel)

S2. List of 1,268 search results curated from literature search (Excel)

S3. LCR22 recombination rate data

S4. Logistic regression data for 1,977 CNVs (Excel)

S5. Demographic data for 3q29 cohort

S6. Summarized data for logistic regression with deletions only

S7. Summarized data for logistic regression with duplications only

S8. Sensitivity analysis results for linear regression analysis with deletions and duplications combined

S9: Predicted paternal origin probability for loci with small sample sizes or missing from analysis

**Supplemental Materials and Methods**

**Supplemental References**

**SUPPLEMENTAL FIGURES**

**Figure S1. Schematic of recombination rate calculations.** The recombination rate (cM/Mb) data from deCODE is publicly available as recombination rates estimated for variably-sized physical intervals bounded by two SNP markers (red and blue rulers). A representative image of the 3q29 locus is shown. Raw male (blue) and female (red) recombination rates are summarized and binned to demonstrate differences in male and female rates. Calculations were completed with raw recombination rate data. First, weighted average male and female recombination rates were calculated by weighting the estimated recombination rate within a respective SNP interval by the total number base pairs contained within that SNP interval. Weighted recombination rates were then averaged across the CNV interval for males and females, separately. The ratio of weighted average male and female recombination rates was then calculated for each CNV interval by dividing the weighted average male recombination rate by the weighted average female recombination rate. See Figures S2-S12 for plotted raw male and female recombination rates for all loci included in the current analysis.

**Figure S2. Raw deCODE recombination across 1q21.1 TAR, 1q21.1, 2q13, and 5q35 regions.** Male (blue) and female (red) recombination across the 3q29 canonical regions (shaded area). X-axis is position in Mb. Location of flanking LCRs pulled from UCSC Genome Browser; hg38).

**Figure S2. Raw deCODE recombination across 1q21.1 TAR and 1q21.1 regions.** Male (blue) and female (red) recombination across the canonical 1q21.1 TAR and 1q21.1 regions (black bar). Location of flanking LCRs pulled from UCSC Genome Browser; hg38 (hatched bars). X-axis is position along the chromosome in Mb. Y-axis is the scaled probability of recombination (cM) across the interval. The curves summarize the rate of increase in probability of recombination over the interval. The ratio of the right-most y-values of the male and female curves roughly equals the male-to-female recombination rate ratio.

**Figure S3. Raw deCODE recombination across 2q13 and 3q29 regions.** Male (blue) and female (red) recombination across the canonical 2q13 and 3q29 regions (black bar). Location of flanking LCRs pulled from UCSC Genome Browser; hg38 (hatched bars). X-axis is position along the chromosome in Mb. Y-axis is the scaled probability of recombination (cM) across the interval. The curves summarize the rate of increase in probability of recombination over the interval. The ratio of the right-most y-values of the male and female curves roughly equals the male-to-female recombination rate ratio.

**Figure S4. Raw deCODE recombination across 5q35 and 7q11.23 regions.** Male (blue) and female (red) recombination across the canonical 5q35 and 7q11.23 regions (black bar). Location of flanking LCRs pulled from UCSC Genome Browser; hg38 (hatched bars). X-axis is position along the chromosome in Mb. Y-axis is the scaled probability of recombination (cM) across the interval. The curves summarize the rate of increase in probability of recombination over the interval. The ratio of the right-most y-values of the male and female curves roughly equals the male-to-female recombination rate ratio.

**LCRs**

**LCRs**

**Figure S5. Raw deCODE recombination across 8p23.1 and 11q13.2q13.4 regions.** Male (blue) and female (red) recombination across the canonical 8p23.1 and 11q13.2q13.4 regions (black bar). Location of flanking LCRs pulled from UCSC Genome Browser; hg38 (hatched bars). X-axis is position along the chromosome in Mb. Y-axis is the scaled probability of recombination (cM) across the interval. The curves summarize the rate of increase in probability of recombination over the interval. The ratio of the right-most y-values of the male and female curves roughly equals the male-to-female recombination rate ratio.

**Figure S6. Raw deCODE recombination across 15q13.3 and 15q24.** Male (blue) and female (red) recombination across the canonical 15q13.3 and 15q24 regions (black bar). Location of flanking LCRs pulled from UCSC Genome Browser; hg38 (hatched bars). LCRs demarking different 15q24 intervals are denoted with letters (A-E). X-axis is position along the chromosome in Mb. Y-axis is the scaled probability of recombination (cM) across the interval. The curves summarize the rate of increase in probability of recombination over the interval. The ratio of the right-most y-values of the male and female curves roughly equals the male-to-female recombination rate ratio.

**Figure S7. Raw deCODE recombination across 15q25.2 and 16p11.2 regions.** Male (blue) and female (red) recombination across the canonical 15q25.2 and 16p11.2 regions (black bar). Location of flanking LCRs pulled from UCSC Genome Browser; hg38 (hatched bars). X-axis is position along the chromosome in Mb. Y-axis is the scaled probability of recombination (cM) across the interval. The curves summarize the rate of increase in probability of recombination over the interval. The ratio of the right-most y-values of the male and female curves roughly equals the male-to-female recombination rate ratio.

**Figure S8. Raw deCODE recombination across distal 16p11.2 and 16p11.2p12.1 regions.** Male (blue) and female (red) recombination across the canonical distal 16p11.2 and 16p11.2p12.1 regions (black bar). Location of flanking LCRs pulled from UCSC Genome Browser; hg38 (hatched bars). X-axis is position along the chromosome in Mb. Y-axis is the scaled probability of recombination (cM) across the interval. The curves summarize the rate of increase in probability of recombination over the interval. The ratio of the right-most y-values of the male and female curves roughly equals the male-to-female recombination rate ratio.

**Figure S9. Raw deCODE recombination across 16p13.11 and 17p11.2 regions.** Male (blue) and female (red) recombination across the canonical 16p13.11 and 17p11.2 regions (black bar). Location of flanking LCRs pulled from UCSC Genome Browser; hg38 (hatched bars). X-axis is position along the chromosome in Mb. Y-axis is the scaled probability of recombination (cM) across the interval. The curves summarize the rate of increase in probability of recombination over the interval. The ratio of the right-most y-values of the male and female curves roughly equals the male-to-female recombination rate ratio.

**Figure S10. Raw deCODE recombination across 17q11.2 and 17q12 regions.** Male (blue) and female (red) recombination across the canonical 17q11.2 and 17q12 regions (black bar). Location of flanking LCRs pulled from UCSC Genome Browser; hg38 (hatched bars). X-axis is position along the chromosome in Mb. Y-axis is the scaled probability of recombination (cM) across the interval. The curves summarize the rate of increase in probability of recombination over the interval. The ratio of the right-most y-values of the male and female curves roughly equals the male-to-female recombination rate ratio.

**Figure S11. Raw deCODE recombination across 17q21.31 and 17q23.1q23.2 regions.** Male (blue) and female (red) recombination across the canonical 17q21.31 and 17q23.1q23.2 regions (black bar). Location of flanking LCRs pulled from UCSC Genome Browser; hg38 (hatched bars). X-axis is position along the chromosome in Mb. Y-axis is the scaled probability of recombination (cM) across the interval. The curves summarize the rate of increase in probability of recombination over the interval. The ratio of the right-most y-values of the male and female curves roughly equals the male-to-female recombination rate ratio.

**Figure S12. Raw deCODE recombination across 22q11.2 region.** Male (blue) and female (red) recombination across the canonical 22q11.2 region (black bar). Location of flanking LCRs pulled from UCSC Genome Browser; hg38 (hatched bars). LCRs demarking different 22q11.2 intervals are denoted with letters (A-D) X-axis is position along the chromosome in Mb. Y-axis is the scaled probability of recombination (cM) across the interval. The curves summarize the rate of increase in probability of recombination over the interval. The ratio of the right-most y-values of the male and female curves roughly equals the male-to-female recombination rate ratio.

**Figure S13. Logistic regression for deletions.** Estimated (black curve) and observed paternal origin proportions for 1,913 deletions from 22 loci are shown. Curated parent of origin data are collapsed by loci into single data points; plotted recombination rate ratios are the average of the metric for all CNVs within the data point. Datapoint size and color correspond to the number of CNVs collapsed into the data point. Recombination rates predict parent of origin for deletions mediated by NAHR. *p*=8.88x10^-14^, β=0.6721, CI_95%_=(0.5009,0.8546).

**Figure S14. Logistic regression for duplications.** Estimated (black curve) and observed paternal origin proportions for 64 duplications from 11 loci are shown. Curated parent of origin data are collapsed by loci into single data points; plotted recombination rate ratios are the average of the metric for all CNVs within the data point. Datapoint size and color correspond to the number of CNVs collapsed into the data point. Recombination rates predict parent of origin for deletions mediated by NAHR. *p*=0.02, β=0.8304, CI_95%_=(0.1508,1.6017).

**Figure S15. Linear regression with combined CNV parent of origin data.** Log_e_-transformed male to female parental origin ratio regressed on log_e_-transformed average male to female recombination rate ratio. Curated parent of origin data from loci with >10 samples are collapsed by loci into single data points; plotted recombination rate ratios are the average of the metric for all CNVs within the data point. Datapoint size and color correspond to the number of CNVs collapsed into the data point. Recombination rates are associated with male-to-female parental origin ratios for CNVs mediated by NAHR (multiple r^2^=0.8512, *p*=0.001, β=0.9540, CI_95%_=(0.5555,1.3525)). This estimate is not influenced by any particular data point as demonstrated by a sensitivity analysis (Table S7).

**SUPPLEMENTAL TABLES**

| **Table S3. LCR22 Recombination rate data** | | | | | | |
| --- | --- | --- | --- | --- | --- | --- |
| **LCR Interval** | **Begin** | **End** | **Pop. Frequency** | **^b^Avg. Male Recombination Rate(1)** | **^b^Avg. Female Recombination Rate(1)** | **^c^M:F Recombination Ratio(1)** |
| 22q11.2 AB | 18872532 | 20326091 | 0.050 | 1.28300587 | 3.42708495 | 0.37437236 |
| 22q11.2 AC | 18872532 | 20702815 | 0.025 | 1.52325126 | 3.61442402 | 0.42143679 |
| 22q11.2 AD | 18872532 | 21337106 | 0.850 | 1.36673167 | 3.44873357 | 0.39629958 |
| 22q11.2 BC | 20326091 | 20702815 | 0.025 | 2.45023972 | 4.33727307 | 0.56492632 |
| 22q11.2 BD | 20326091 | 21337106 | 0.025 | 1.48712659 | 3.47986356 | 0.42735198 |
| 22q11.2 CD | 20702815 | 21337106 | 0.025 | 0.91497120 | 2.97050338 | 0.30801891 |
| Recombination rate calculations for chr 22q11.2 LCR intervals. Begin and End coordinates curated from UCSC Genome Browser, hg38. ^a^Male to female CNV parent of origin counts. ^b^Average male and female recombination rates are as described in Table 2. ^c^22q11.2 weighted average was calculated by weighting the M:F recombination rate ratio for each interval by the interval population frequency. | | | | | | |

| **Table S5. Demographic data for 3q29 cohort and parental origin of the 3q29 deletion** | | | | | | | |
| --- | --- | --- | --- | --- | --- | --- | --- |
| **Family ID** | **Subject ID** | **^a^Father ID** | **^a^Mother ID** | **Sex** | **^b^Pat:Mat MEs** | **Deletion Parental Origin** | **^c^Parent of Origin Age** |
| 93315602 | 834-3156-1031 | N/A | 834-3156-2096 | F | --:0 | Pat | 24y |
| 93315602 | 834-3156-2096 |  |  | F |  |  |  |
| 93316202 | 834-3162-1031 | N/A | 834-3162-2096 | F | --:0 | Pat | 40y |
| 93316202 | 834-3162-2096 |  |  | F |  |  |  |
| 93316402 | 834-3164-1001 | 834-3164-2046 | 834-3164-2096 | M | 37:0 | Pat | 32y |
| 93316402 | 834-3164-2046 |  |  | M |  |  |  |
| 93316402 | 834-3164-2096 |  |  | F |  |  |  |
| 93316802 | 834-3168-1001 | 834-3168-2046 | 834-3168-2096 | M | 36:0 | Pat | 43y |
| 93316802 | 834-3168-2046 |  |  | M |  |  |  |
| 93316802 | 834-3168-2096 |  |  | F |  |  |  |
| 93318102 | 834-3181-1001 | 834-3181-2046 | 834-3181-2096 | M | 44:0 | Pat | 34y |
| 93318102 | 834-3181-2046 |  |  | M |  |  |  |
| 93318102 | 834-3181-2096 |  |  | F |  |  |  |
| 93318902 | 834-3189-1001 | 834-3189-2046 | 834-3189-2096 | M | 67:0 | Pat | 29y |
| 93318902 | 834-3189-2046 |  |  | M |  |  |  |
| 93318902 | 834-3189-2096 |  |  | F |  |  |  |
| 93322602 | 834-3226-1031 | 834-3226-2046 | 834-3226-2096 | F | 0:40 | Mat | 41y |
| 93322602 | 834-3226-2046 |  |  | M |  |  |  |
| 93322602 | 834-3226-2096 |  |  | F |  |  |  |
| 93324602 | 834-3246-1001 | 834-3246-2046 | 834-3246-2096 | M | 44:0 | Pat | 38y |
| 93324602 | 834-3246-2046 |  |  | M |  |  |  |
| 93324602 | 834-3246-2096 |  |  | F |  |  |  |
| 93325702 | 834-3257-1031 | 834-3257-2046 | 834-3257-2096 | F | 36:0 | Pat | 38y |
| 93325702 | 834-3257-2046 |  |  | M |  |  |  |
| 93325702 | 834-3257-2096 |  |  | F |  |  |  |
| 93327702 | 834-3277-1001 | 834-3277-2046 | 834-3277-2096 | M | 54:0 | Pat | 28y |
| 93327702 | 834-3277-2046 |  |  | M |  |  |  |
| 93327702 | 834-3277-2096 |  |  | F |  |  |  |
| 93339602 | 834-3396-1031 | 834-3396-2046 | 834-3396-2096 | F | 33:0 | Pat | 43y |
| 93339602 | 834-3396-2046 |  |  | M |  |  |  |
| 93339602 | 834-3396-2096 |  |  | F |  |  |  |
| 93342002 | 834-3420-1001 | 834-3420-2046 | 834-3420-2096 | M | 27:0 | Pat | 36y |
| 93342002 | 834-3420-2046 |  |  | M |  |  |  |
| 93342002 | 834-3420-2096 |  |  | F |  |  |  |
| 93320602 | 834-3206-1031 | 834-3206-2096 | 834-3206-2046 | F | ^d^401:20 | Pat | 32y |
| 93320602 | 834-3206-2046 |  |  | M |  |  |  |
| 93320602 | 834-3206-2096 |  |  | F |  |  |  |
| 93314702 | 834-3147-1001 | 834-3147-2046 | 834-3147-2096 | M | ^d^743:22 | Pat | 29y |
| 93314702 | 834-3147-2046 |  |  | M |  |  |  |
| 93314702 | 834-3147-2096 |  |  | F |  |  |  |
| Demographic data is self-reported. ^a^N/A indicates information is not available. Grandparental samples were not collected. ^b^Number of informative SNPs per parent supporting parental origin given in the order father:mother; -- indicates parent unavailable. ^c^Age of parent of origin corresponds to parent's age at birth of affected child. ^d^Family 3206 and 3147 parental origin determined with whole-genome sequencing data. | | | | | | | |

| **Table S6. Summary of deletions grouped by locus, parental origin, and recombination data** | | | | | | |
| --- | --- | --- | --- | --- | --- | --- |
| **Locus** | **BED Coordinates(2)** | **# Samples (%)** | **^a^M:F Origin Counts** | **^b^Avg. Male Recombination Rate(1)** | **^b^Avg. Female Recombination Rate(1)** | **^c^Log_e_ M:F Recombination Ratio(1)** |
| 1q21.1 | chr1:147101794-147921262 | 7 (0.37%) | 4:3 | 0.12887809 | 0.53120997 | -1.4162905 |
| 1q21.1 TAR | dchr1:145686999-146048495 | 1 (0.05%) | 1:0 | 0.15712388 | 0.77814863 | -1.599883 |
| 3q29 | chr3:195988732-197628732 | 22 (1.15%) | 21:1 | 3.1305211 | 0.27775988 | 2.42219776 |
| 5q35 | chr5:176290391-177630393 | 41 (2.14%) | 36:5 | 1.29955355 | 0.97941355 | 0.28282209 |
| 7q11.23 | chr7:73328061-74727726 | 598 (31.26%) | 287:311 | 0.49353298 | 1.92655808 | -1.3619006 |
| 8p23.1 | chr8:8235068-12035082 | 1 (0.05%) | 1:0 | 0.67201752 | 1.81857951 | -0.9955266 |
| 11q13.2q13.4 | chr11:67985953-71571306 | 1 (0.05%) | 0:1 | 0.8431765 | 2.23501635 | -0.9748275 |
| 15q13.3 | chr15:30840505-32190507 | 5 (0.26%) | 4:1 | 1.63838993 | 1.90155369 | -0.1489573 |
| 15q24 AC | chr15:72670606-75240606 | 1 (0.05%) | 1:0 | 0.28479919 | 0.86129537 | -1.1066532 |
| 15q24 AD | chr15:72670606-75720604 | 3 (0.16%) | 1:2 | 0.27613544 | 0.82152961 | -1.0902765 |
| 15q24 BD | chr15:73720606-75720604 | 1 (0.05%) | 0:1 | 0.30739967 | 0.68432207 | -0.8002799 |
| 15q24 BE | chr15:73720606-77840603 | 2 (0.10%) | 0:2 | 0.23485125 | 0.72623183 | -1.128917 |
| 15q25.2 | chr15:82513967-84070244 | 5 (0.26%) | 0:5 | 0.21225081 | 0.32633295 | -0.4301495 |
| 16p11.2 | chr16:29641178-30191178 | 79 (4.13%) | 9:70 | 0.06565904 | 1.28716751 | -2.9757241 |
| 16p11.2 distal | chr16:28761178-29101178 | 3 (0.16%) | 0:3 | 0.11421814 | 1.52707077 | -2.5929965 |
| 16p13.11 | chr16:15408642-16198642 | 1 (0.05%) | 1:0 | 1.66089552 | 2.45240302 | -0.3897114 |
| 17p11.2 | chr17:16805961-20576095 | 59 (3.08%) | 35:24 | 0.1888066 | 1.19115966 | -1.8419594 |
| 17q11.2 | chr17:30838856-31888868 | 62 (3.24%) | 10:52 | 0.26024285 | 1.85442774 | -1.9637162 |
| 17q12 | chr17:36460073-37846263 | 6 (0/31%) | 4:2 | 0.64750654 | 3.64754421 | -1.7286805 |
| 17q21.31 | chr17:45626851-46106851 | 35 (1.83%) | 18:17 | 0.38234179 | 0.98304273 | -0.9443376 |
| 17q23.1q23.2 | chr17:59987857-62227857 | 2 (0.10%) | 0:2 | 0.56466054 | 1.30765625 | -0.839767 |
| 22q11.2 | chr22:18924718-21111383 | 978 (51.12%) | 411:567 | 1.45946494 | 3.69205976 | -0.9281146 |
| **All** | **—** | **1913 (100%)** | **844:1069** | **—** | **—** | **—** |
| Summarized duplication data. Data are consolidated by locus. BED coordinates correspond to hg38 (LiftOver from hg18 coordinates in Coe et al., 2014). ^a^Male to female CNV parent of origin counts. ^b^Average male and female recombination rates are as described in Table 2. ^c^Natural log-transformed average male to female recombination rate ratio for the locus | | | | | | |

| **Table S7. Summary of duplications grouped by locus, parental origin, and recombination data** | | | | | | |
| --- | --- | --- | --- | --- | --- | --- |
| **Locus** | **BED Coordinates(2)** | **# Samples (%)** | **^a^M:F Origin Counts** | **^b^Avg. Male Recombination Rate(1)** | **^b^Avg. Female Recombination Rate(1)** | **^c^Log_e_ M:F Recombination Ratio(1)** |
| 1q21.1 | chr1:147101794-147921262 | 2 (3.13%) | 2:0 | 0.1038527 | 0.42854444 | -1.4174209 |
| 2q13 | chr2:110625954-112335952 | 1 (1.56%) | 1:0 | 0.44854539 | 1.64377881 | -1.2987431 |
| 7q11.23 | chr7:73328061-74727726 | 20 (31.25%) | 9:11 | 0.49361225 | 1.92693374 | -1.361935 |
| 8p23.1 | chr8:8235068-12035082 | 2 (3.13%) | 0:2 | 0.67201752 | 1.81857951 | -0.9955266 |
| 15q13.3 | chr15:30840505-32190507 | 1 (1.56%) | 1:0 | 1.62649391 | 1.88629391 | -0.1481873 |
| 16p11.2 | chr16:29641178-30191178 | 19 (29.69%) | 2:17 | 0.06591856 | 1.28839583 | -2.9727332 |
| 16p11.2 distal | chr16:28761178-29101178 | 1 (1.56%) | 0:1 | 0.14338352 | 1.88529263 | -2.5763154 |
| 16p11.2p12.1 | chr16:21341178-29431178 | 1 (1.56%) | 1:0 | 0.5534655 | 2.68382469 | -1.5787988 |
| 16p13.11 | chr16:15408642-16198642 | 1 (1.56%) | 0:1 | 1.68055204 | 2.47808756 | -0.3883648 |
| 17p11.2 | chr17:16805961-20576095 | 12 (18.75%) | 9:3 | 0.1888066 | 1.19115966 | -1.8419594 |
| 17q21.31 | chr17:45626851-46106851 | 4 (6.25%) | 1:3 | 0.38234179 | 0.98304273 | -0.9443376 |
| **All** | **—** | **64 (100%)** | **26:38** | **—** | **—** | **—** |
| Summarized duplication data. Data are consolidated by locus. BED coordinates correspond to hg38 (LiftOver from hg18 coordinates in Coe et al., 2014). ^a^Male to female CNV parent of origin counts. ^b^Average male and female recombination rates are as described in Table 2. ^c^Natural log-transformed average male to female recombination rate ratio for the locus | | | | | | |

| **Table S8. Sensitivity analysis results for linear regression analysis with deletions and duplications combined** | | | | |
| --- | --- | --- | --- | --- |
| **Locus Removed** | **CNV Type** | **^a^r^2^** | ***p*-value** | **Beta** |
| None | Deletion/Duplication | 0.8512 | 0.0011 | 0.9540 |
| 3q29 | Deletion | 0.7221 | 0.0155 | 0.9823 |
| 5q35 | Deletion | 0.8585 | 0.0027 | 0.8991 |
| 7q11.23 | Deletion/Duplication | 0.8906 | 0.0014 | 0.9719 |
| 16p11.2 | Deletion/Duplication | 0.7862 | 0.0078 | 0.9154 |
| 17p11.2 | Deletion/Duplication | 0.9042 | 0.0010 | 0.9729 |
| 17q11.2 | Deletion | 0.8597 | 0.0026 | 0.9204 |
| 17q21.31 | Deletion/Duplication | 0.8543 | 0.0029 | 0.9446 |
| 22q11.2 | Deletion | 0.9061 | 0.0009 | 0.9785 |
| ^a^Multiple r^2^ value as reported by R (3). | | | | |

| **Table S9. Predicted probability of paternal origin for loci with N < 10** | | | |
| --- | --- | --- | --- |
| **Locus** | **BED Coordinates** | **Sample (N)** | **Pred. Paternal Probability** |
| 1q21.1 | chr1:147101794-147921262 | 9 | 0.39899 |
| 1q21.1 TAR | chr1:145686999-146048495 | 1 | 0.37028 |
| 2q11.2 | chr2:96060525-97010536 | 0 | 0.37428 |
| 2q11.2q13 | chr2:100077106-107827112 | 0 | 0.53549 |
| 2q13 | chr2:110625954-112335952 | 1 | 0.41771 |
| 7q11.23 distal | chr7:75332889-77032747 | 0 | 0.36502 |
| 7q11.23 proximal | chr7:67017578-72805248 | 0 | 0.47948 |
| 8p23.1 | chr8:8235068-12035082 | 3 | 0.46711 |
| 10q23 | chr10:80200264-87040263 | 0 | 0.53914 |
| 11q13.2q13.4 | chr11:67985953-71571306 | 1 | 0.47051 |
| 15q13.3 | chr15:30840505-32190507 | 6 | 0.60525 |
| 15q24 AC | chr15:72670606-75240606 | 1 | 0.44889 |
| 15q24 AD | chr15:72670606-75720604 | 3 | 0.45157 |
| 15q24 BD | chr15:73720606-75720604 | 1 | 0.44054 |
| 15q24 BE | chr15:73720606-77840603 | 2 | 0.44525 |
| 15q25.2 | chr15:82513967-84070244 | 5 | 0.56021 |
| 15q25.2 Cooper | chr15:84595765-85155765 | 0 | 0.44832 |
| 16p11.2 distal | chr16:28761178-29101178 | 4 | 0.23931 |
| 16p11.2p12.1 | chr16:21341178-29431178 | 0 | 0.37354 |
| 16p11.2p12.2 | chr16:21601178-29031178 | 1 | 0.37478 |
| 16p12.1 | chr16:21931178-22451178 | 0 | 0.51951 |
| 16p13.11 | chr16:15408642-16198642 | 2 | 0.56638 |
| 17q12 | chr17:36460073-37846263 | 6 | 0.35069 |
| 17q23 | chr17:59577857-59997857 | 0 | 0.60003 |
| 17q23.1q23.2 | chr17:59987857-62227857 | 2 | 0.49424 |
| 22q11.2 distal | chr22:21555711-23307813 | 0 | 0.58724 |
| Probability of paternal origin for loci predicted from combined logistic regression: parental origin ~ log_e_(M:F recombination rate ratio). | | | |

**SUPPLEMENTAL MATERIALS AND METHODS**

**Literature Search and Data Curation**

CNV loci were curated from Coe et al., 2014 (2). This paper is an expansion of Cooper et al., 2011 (4), and includes 55 known CNV loci associated with genomic disorders. We applied a set of exclusion criteria to the 55 loci in order to filter for loci in which CNVs are flanked by LCRs, *i.e.* mediated by NAHR. As individuals with CNV at imprinted loci are generally ascertained by phenotype, and the phenotype is determined by the parent of origin, imprinted loci would introduce an ascertainment bias to the analysis and were therefore excluded. Loci were determined to be imprinted if the canonical interval overlapped imprinted genes as indicated by the Geneimprint database ([www.geneimprint.com](http://www.geneimprint.com)). In total, 17 loci were excluded from further analysis because the loci were not flanked by LCRs and/or the loci were imprinted (Additional File 2: Table S1). For the remaining 38 loci, we conducted a systematic PubMed literature search for studies that reported parental origin of CNVs at these loci. On PubMed, loci were searched using a phrase with the format: *cytogenic locus OR syndrome.* The number of results/hits was recorded and if the initial search produced more than 100 hits, a sub-search was performed. This sub-search used the following format: *(cytogenic locus OR syndrome) + parental bias OR parental origin OR transmission bias OR parent-of-origin OR parent of origin OR maternal bias OR paternal bias OR paternal origin OR maternal origin).*

Studies were included in analysis:

- If the study reported parental origin
- If the authors of the study adequately interrogated the patients for the presence of the CNV(s). This included stating that the CNV(s) was previously or currently confirmed, and/or a confirmation via FISH, aCGH, marker PCR, SNP array, whole-genome sequencing, etc.
- If the authors of the study stated the CNVs were *de novo*.
- CNVs in monozygotic twins were clearly treated as the result of a single meiotic event. MZ twins are the product of one egg fertilized with one sperm, that then splits into two zygotes, thus the CNV present originated in one of the *single* gametes.

We note that 17q11.2q12, a known genomic disorder locus associated with Charcot-Marie-Tooth disease type 1A (CMT1A; duplication) and hereditary neuropathy with liability to pressure palsies (HNPP; deletion), is mediated by NAHR, and thus applicable for inclusion in our analysis. However, subsequent research on the locus produced reports of a sex-dependent bias in both the mechanism for the formation of the associated CNVs and the resulting phenotype (5, 6). CNVs of paternal origin are generated via NAHR between homologous chromosomes during meiosis and are largely duplications (resulting in CMT1A), whereas CNVs of maternal origin are produced via intrachromosomal rearrangement between sister chromatids and result in equal numbers of deletions and duplications (resulting in CMT1A and HNPP). This is likely to cause a complex ascertainment bias and introduce a confounder associated with this locus. For this reason, we excluded the 17q11.2q12 locus from this study.

77 studies in total satisfied inclusion criteria and included parental origin data for 24 loci encompassing 1,977 deletion and duplication events (Table 1 and Table 2). All search phrases and studies curated as part of the current analysis and the loci used in the logistic regression analysis are listed in Additional file 2: Table S1, Additional file 3: Table S2 and Additional File 4: Table S4, respectively.

**Study Subject Recruitment**

Individuals with a clinically confirmed diagnosis of 3q29 deletion were ascertained through the internet-based 3q29 registry (https://3q29deletion.patientcrossroads.org/) as previously described (7, 8). We obtained blood samples and determined parental origin of the 3q29 deletion in 14 families. Of the 14 families, 12 were full trios. The remaining two families were both mother and child pairs.

**Sample Collection and Banking**

Whole blood was collected from proband, mother, and father as previously reported (8) and banked at the NIMH Repository and Genomics Resource (NRGR; Piscataway, New Jersey, USA).

**DNA Isolation**

DNA samples were isolated from/obtained from biobanked samples at the NIMH Repository and Genomics Resource (NRGR; Piscataway, New Jersey, USA). The source of DNA was either whole blood or LCLs derived from biobanked blood samples.

**SNP Genotyping and QC**

SNP genotyping was performed on 12 of the 14 families (10 full trios, 2 mother-child pairs) by AKESOgen (Peachtree Corners, Georgia, USA) on the Illumina GSA-24 v 3.0 array, which contains 654,027 SNPs genome-wide. DNA from participants was normalized and genotyped according to AKESOgen/Illumina protocols. Data was returned as separate final reports that were combined into one deduplicated final report and converted into PLINK format for quality control (QC). QC was performed with PLINK 1.9 (9). Briefly, indel calls, unmappable SNPs, and SNPs with call rates less than 97% were dropped from the SNP call set (n = 35,187), leaving 611,986 SNPs genome-wide. Samples’ reported sex and family relationships were verified using this set of quality SNPs and PLINK 1.9 (9). F coefficient estimates for the X chromosome were calculated and sex assignment was inferred for each sample in the batch. Before sex was inferred, the *--split-x* flag with the *hg38* modifier was used to identify pseudoautosomal regions of the X chromosomes for subsequent removal during the sex check. The default parameters for the *--check-sex* flag were used to infer sex. A sample with an F coefficient less than or equal to 0.2 was assigned as female, and a sample with an F coefficient greater than or equal to 0.9 was assigned as male. Any samples with an opposite sex assignment than indicated by the given pedigree were flagged and investigated for possible sample swapping or sample mixture. Expected relationships between related samples of the batch were verified with PLINK 1.9 (9). Variants were LD-pruned using the *--indep-pairwise* flag using 50, 5, and 0.2 for the variant count window size, variant count step size, and r^2^, respectively. The *--genome* flag was used to infer relationships (coefficient of relatedness; *r*) on this set of pruned SNPs. . All samples' sex information and relationship information were concordant with our expectation based on information provided by the families.

**Whole-genome Sequencing**

For 2 full trios (families 3206 and 3147; 6 samples), parent of origin was determined from whole-genome sequence data. All samples were sequenced at the Hudson–Alpha Institute of Biotechnology (Birmingham, Alabama, USA) using their published protocols. Sequencing was performed to approximately 30X coverage per genome on the Illumina NovaSeq 6000 platform. Following sequencing, all base-calling was performed using standard Illumina software to generate the final FASTQ files for each sample.

**Sequence Alignment: PEMapper**

FASTQ files were aligned on a per-sample basis with PEMapper[9] using default parameters and a Smith-Waterman alignment threshold of 95%, as recommended for 150-bp paired-end reads. Alignment was performed relative to the human Hg38 reference as reported by the University of California at Santa Cruz (UCSC) Genome Browser on July 1, 2015. The output from PEMapper (10), pileup, and indel files were used as input for variant calling with PECaller (10). Pileup files contained the number of reads where an A, C, G, or T nucleotide was seen together with the number of times that base appeared deleted or there was an insertion immediately after the base. Indel files contained the nucleotide sequence of the deletions and insertions indicated in the pileup files. Alignment performance was checked before moving to variant calling. No samples were removed based on failed sequence alignment.

**Variant Calling: PECaller**[9]

Variant calling was performed in a single batch using PECaller (10), which assumes multiple samples all done on the same technology will be available. Optimal PECaller[9] performance is achieved when at least 50 genomes are called in batch; 57 control genomes were included with the genomes from families 3206 and 3147 (63 genomes total). PECaller (10) was run with the default theta value of 0.001 and a 95% posterior probability for a genotype to be considered called. A posterior probability of less than 95% was considered a missing call. Calls were produced for the repeat-masked (unique) subset of the human Hg38 reference as reported by the University of California at Santa Cruz (UCSC) Genome Browser on July 1, 2015. The initial .snp file output from PECaller[9] was used in a subsequent step to merge SNP variant calls with INDEL variant calls, producing a final “merged” .snp file. This raw file was used for site and sample quality control.

**Whole-genome Sequence Quality Control**

Quality control was performed on a per-site and per-sample basis. The following metrics were used to flag and/or exclude samples and variant sites from QC and analysis, and were calculated using a custom QC pipeline consisting of multiple in-house-developed scripts, PLINK 1.9 (9), R (3), and Bystro (11):

1. *Per-site QC: Missing call rate:* The missing call rates for variant sites were calculated as described above. Variants with a missing call rate greater than or equal to 10% were removed from subsequent QC *and* variant analysis.
2. *Sample Mixture Check:* Possible sample mixture was checked by calculating the ratios of minor allele homozygous calls to heterozygous calls. This number varies between call batches, and thus cannot be compared across different calling experiments. However, non-mixed samples within the same calling batch should exhibit similar ratios. The ratios were calculated using Bystro (11). No samples were removed based on possible sample mixture.
3. *Per-sample QC: Transition:Transversion Ratio:* Transition:transversion (Ti:Tv) ratios were calculated for each genome in the variant calling batch using a script developed in-house Bystro (11). Based on population expectations, the Ti:Tv ratio for an individual genome is expected to be approximately 2.00, with a ratio of 2.04 representing a quality genome. The batch mean Ti:Tv ratio were calculated using Bystro (11). The control genomes used in batch calling were previously validated for calling performance, therefore a *mean* Ti:Tv ratio less than 2.00 suggests a failed variant-calling experiment. As such, the entire sample batch is resubmitted for variant calling. No samples were removed from analysis on the basis of Ti:Tv ratio.
4. *Per-sample QC: Silent:Replacement Ratio:* Silent:replacement (sil:rep) ratios were calculated for each genome in the variant calling batch using Bystro (11). The expected sil:rep ratio for a single genome is expected to fall between 1.05 and 1.15, with 1.15 indicating a quality genome. The batch mean sil:rep ratio and standard deviation were calculated using Bystro (11). A mean sil:rep less than 1.05 suggested a failed variant-calling experiment and the sample batch was resubmitted for variant calling. Any samples with a sil:rep ratio less than 1.05 were flagged and removed from subsequent QC. No samples were removed from analysis on the basis of sil:rep ratio.
5. *Per-sample QC: Missing call rate:* The missing call rates for samples were calculated using PLINK (9). The merged .snp file generated after the indel merging process was converted to a VCF [v4.0] format (snp_to_vcf2), the appropriate VCF headers were appended to the file, and multiallelic variants were split using BCFtools 1.3 (12) before the final BCF was loaded into PLINK (9). The following flags were used during loading: *--bcf*, and *--keep-allele-order*. Per sample missing call rates were calculated using the *–missing* flag in PLINK (9), and the batch mean missing call rate and standard deviation were calculated using R (3). A mean missing call rate greater than or equal to 3% indicated a failed variant-calling experiment and the sample batch was resubmitted for variant calling. No samples in the current analysis were removed based on low call rate.
6. *Sex Check:* PLINK was used to calculate the F coefficient estimates for the X chromosome and impute sex assignment for each sample in the batch. Before sex was inferred, the *--split-x* flag with the *hg38* modifier was used to identify pseudoautosomal regions of the X chromosomes for subsequent removal during the sex check. The default parameters for the *--check-sex* flag were used to infer sex. A sample with an F coefficient less than or equal to 0.2 was assigned as female, and a sample with an F coefficient greater than or equal to 0.9 was assigned as male. All samples' inferred sex matched our expectations based on provided information.
7. *Relationship Inference:* Expected relationships between related samples of the batch were verified with PLINK. Variants were LD-pruned using the *--indep-pairwise* flag using 50, 5, and 0.2 for the variant count window size, variant count step size, and r^2^, respectively. The *--genome* flag was used to infer relationships (coefficient of relatedness; *r*) on this set of pruned SNPs. Among the control genomes, there was a known parent-offspring relationship, which was used as a positive control, while the remaining control genomes were known to be unrelated. All samples' inferred relationships matched our expectations based on information provided by the families.

**Parental Origin Analysis**

Parental origin of the 3q29 deletion was determined for 12 trios --10 full trios and 2 trios for which only the child and mother’s info was available -- using SNP array data. Briefly, using PLINK 1.9 (9), 404 SNPs located within the 3q29 deletion interval (chr3:196029182-197617792; hg38) were isolated for analysis. Mendelian errors (MEs) were called for these SNPs using PLINK’s *--mendel* function with the *-duos* modifier to also call MEs for the mother-daughter pairs. The parent with the most mendelian errors was considered the parent of origin for the 3q29 deletion. Parental origin was determined using WGS data for two trios (3147 and 3206). Briefly, variants in the 3q29 critical region were called using PECaller. The variants with a sample minor allele frequency (MAF) less than 10% were filtered from this set of SNPs, and MEs were called. As in the SNP array analysis, the parent with the most MEs was considered the parent of origin for the 3q29 deletion

**Paternal Age Analysis**

Age of fathers at birth data for ~3 million U.S. births in 2018 (latest data available) were obtained from the National Center for Health Statistics (NCHS) (<https://www.cdc.gov/nchs/index.htm>). The mean age of parents in our 3q29 cohort was collected from self-reported data in conjunction with the Emory University 3q29 project (<http://genome.emory.edu/3q29/>) and compared to the U.S. average via a two-tailed two-sample t-test using R (3).

**Breakpoint Usage Determination**

We calculated average male and recombination rates over CNV intervals as determined by reported or canonical breakpoints (2). When possible, we used breakpoints reported by the authors of the studies to calculate average male and female recombination rates and used the UCSC LiftOver tool to convert the breakpoints to hg38. To reduce the possibility of the failure of breakpoints reported on older human genome builds to successfully liftover to hg38, we used breakpoints reported by the authors only if they were reported on human genome build 19 (hg19) or higher. Otherwise, the breakpoints cited by Coe et al., 2014 were used. We note that each interval from Coe et al., 2014, except the 1q21.1 TAR, locus successfully translated to hg38 coordinates with UCSC LiftOver. For 1q21.1 TAR, we used canonical breakpoints cited in the Clinical Genome Resource (ClinGen) (13). We also note the breakpoints cited in Coe et al., 2014 for the 22q11.2 region correspond to the ~1.5 Mb LCR22A-LCR22B CNV interval and not the more common ~3 Mb LCR22A-LCR22D CNV interval. Where possible to distinguish between the 1.5 Mb and 3.0 Mb 22q11.2 CNVs, we used the appropriate ~1.5 Mb or ~3.0 Mb breakpoints cited in ClinGen (13).

**Calculation of Recombination Rates and Ratios**

Chromosome male and female recombination rates (cM/Mb) were obtained from the deCODE sex-specific maps (1). The recombination rate (cM/Mb) data from deCODE is publicly available as recombination rates calculated for variably-sized physical genomic intervals bounded by two SNP markers. Therefore, for our calculation of the average male and female recombination rates, each bounded recombination rate was weighted by the total number base pairs contained within the respective SNP marker interval. Weighted rates were then averaged across the CNV interval (See Breakpoint Usage below) for males and females, separately. The ratio of weighted average male and female recombination rates was then calculated for each CNV interval by dividing the weighted average male recombination rate by the weighted average female recombination rate. To account for slight differences in the recombination ratios calculated for the different LCR22 intervals at the 22q11.2 locus we used an adjusted recombination ratio composed of the weighted recombination rate ratios calculated for each LCR22 interval. Weights were based on the estimated population prevalence of the different 22q11.2 deletion intervals (Additional file 1: Table S3) (14). The data from deCODE is presented as binned rates across separate chromosomes. As such, each binned recombination rate was weighted by the total base pairs of CNV contained within the respective bin (breakpoints cited in Coe et al, 2014 (2)). Weighted binned rates were then averaged across the CNV interval.

**Logistic Regression Analysis**

Parental origin data was curated for CNVs at the 24 CNV loci from 77 independent studies; only independent samples were included in the analysis (duplicate or overlapping samples were removed). For each CNV locus the male to female recombination rate ratio was calculated as described above. A logistic regression model was fitted to the data with the log_e_-transformed male to female recombination rate ratio as the predictor and parental origin (paternal vs. maternal) as the response variable using R (3). We performed a secondary analysis stratified by deletions/duplications. See Table 2 and Additional File 4: Table S4 for the data calculated and used in the logistic regression.

**Linear Regression Analysis**

Locus-specific estimates for parental origin were derived by combining the data from all published studies for a given locus. To alleviate the uncertainty introduced by small sample sizes, only those loci with more than 10 observations were included. The log_e_-transformed combined male to female parental origin count ratios for each locus was regressed on the calculated averaged log_e_-transformed average male to female recombination rate ratio for that locus’ CNV interval using R (3). Each locus was weighted based on its sample size. A combined analysis (deletions and duplications) was performed under the assumption that an NAHR event produces reciprocal deletion and duplication products, formation of both types of CNVs would be subject to the same biological forces. Thus, for each locus, duplications and deletions were treated equally and grouped under one locus.

**Sensitivity Analysis**

A sensitivity analysis was conducted for the combined linear regression by iteratively running the linear model in R[2]. On each iteration, one data point was removed from the model in order to identify potential influencing points. Results from the analysis are listed in Table S8.

**SUPPLEMENTAL REFERENCES**

1. Halldorsson BV, Palsson G, Stefansson OA, Jonsson H, Hardarson MT, Eggertsson HP, et al. Characterizing mutagenic effects of recombination through a sequence-level genetic map. Science. 2019;363(6425):eaau1043.

2. Coe BP, Witherspoon K, Rosenfeld JA, van Bon BW, Vulto-van Silfhout AT, Bosco P, et al. Refining analyses of copy number variation identifies specific genes associated with developmental delay. Nat Genet. 2014;46(10):1063-71.

3. Team RC. R: A Language and Environment for Statistical Computing. Vienna, Austria2014.

4. Cooper GM, Coe BP, Girirajan S, Rosenfeld JA, Vu TH, Baker C, et al. A copy number variation morbidity map of developmental delay. Nat Genet. 2011;43(9):838-46.

5. Lopes J, Ravise N, Vandenberghe A, Palau F, Ionasescu V, Mayer M, et al. Fine mapping of de novo CMT1A and HNPP rearrangements within CMT1A-REPs evidences two distinct sex-dependent mechanisms and candidate sequences involved in recombination. Hum Mol Genet. 1998;7(1):141-8.

6. Lopes J, Vandenberghe A, Tardieu S, Ionasescu V, Levy N, Wood N, et al. Sex-dependent rearrangements resulting in CMT1A and HNPP. Nat Genet. 1997;17(2):136-7.

7. Glassford MR, Rosenfeld JA, Freedman AA, Zwick ME, Mulle JG, Unique Rare Chromosome Disorder Support G. Novel features of 3q29 deletion syndrome: Results from the 3q29 registry. Am J Med Genet A. 2016;170A(4):999-1006.

8. Murphy MM, Lindsey Burrell T, Cubells JF, Espana RA, Gambello MJ, Goines KCB, et al. Study protocol for The Emory 3q29 Project: evaluation of neurodevelopmental, psychiatric, and medical symptoms in 3q29 deletion syndrome. BMC Psychiatry. 2018;18(1):183.

9. Chang CC, Chow CC, Tellier LC, Vattikuti S, Purcell SM, Lee JJ. Second-generation PLINK: rising to the challenge of larger and richer datasets. Gigascience. 2015;4:7.

10. Johnston HR, Chopra P, Wingo TS, Patel V, International Consortium on B, Behavior in 22q11.2 Deletion S, et al. PEMapper and PECaller provide a simplified approach to whole-genome sequencing. Proc Natl Acad Sci U S A. 2017;114(10):E1923-E32.

11. Kotlar AV, Trevino CE, Zwick ME, Cutler DJ, Wingo TS. Bystro: rapid online variant annotation and natural-language filtering at whole-genome scale. Genome Biol. 2018;19(1):14.

1. Halldorsson BV, Palsson G, Stefansson OA, Jonsson H, Hardarson MT, Eggertsson HP, et al. Characterizing mutagenic effects of recombination through a sequence-level genetic map. Science. 2019;363(6425):eaau1043.

2. Coe BP, Witherspoon K, Rosenfeld JA, van Bon BW, Vulto-van Silfhout AT, Bosco P, et al. Refining analyses of copy number variation identifies specific genes associated with developmental delay. Nat Genet. 2014;46(10):1063-71.

3. Team RC. R: A Language and Environment for Statistical Computing. Vienna, Austria2014.

4. Cooper GM, Coe BP, Girirajan S, Rosenfeld JA, Vu TH, Baker C, et al. A copy number variation morbidity map of developmental delay. Nat Genet. 2011;43(9):838-46.

5. Lopes J, Ravise N, Vandenberghe A, Palau F, Ionasescu V, Mayer M, et al. Fine mapping of de novo CMT1A and HNPP rearrangements within CMT1A-REPs evidences two distinct sex-dependent mechanisms and candidate sequences involved in recombination. Hum Mol Genet. 1998;7(1):141-8.

6. Lopes J, Vandenberghe A, Tardieu S, Ionasescu V, Levy N, Wood N, et al. Sex-dependent rearrangements resulting in CMT1A and HNPP. Nat Genet. 1997;17(2):136-7.

7. Glassford MR, Rosenfeld JA, Freedman AA, Zwick ME, Mulle JG, Unique Rare Chromosome Disorder Support G. Novel features of 3q29 deletion syndrome: Results from the 3q29 registry. Am J Med Genet A. 2016;170A(4):999-1006.

8. Murphy MM, Lindsey Burrell T, Cubells JF, Espana RA, Gambello MJ, Goines KCB, et al. Study protocol for The Emory 3q29 Project: evaluation of neurodevelopmental, psychiatric, and medical symptoms in 3q29 deletion syndrome. BMC Psychiatry. 2018;18(1):183.

9. Chang CC, Chow CC, Tellier LC, Vattikuti S, Purcell SM, Lee JJ. Second-generation PLINK: rising to the challenge of larger and richer datasets. Gigascience. 2015;4:7.

10. Johnston HR, Chopra P, Wingo TS, Patel V, International Consortium on B, Behavior in 22q11.2 Deletion S, et al. PEMapper and PECaller provide a simplified approach to whole-genome sequencing. Proc Natl Acad Sci U S A. 2017;114(10):E1923-E32.

11. Kotlar AV, Trevino CE, Zwick ME, Cutler DJ, Wingo TS. Bystro: rapid online variant annotation and natural-language filtering at whole-genome scale. Genome Biol. 2018;19(1):14.

12. Li H. A statistical framework for SNP calling, mutation discovery, association mapping and population genetical parameter estimation from sequencing data. Bioinformatics. 2011;27(21):2987-93.

13. Rehm HL, Berg JS, Brooks LD, Bustamante CD, Evans JP, Landrum MJ, et al. ClinGen--the Clinical Genome Resource. N Engl J Med. 2015;372(23):2235-42.

14. McDonald-McGinn DM, Hain HS, Emanuel BS, Zackai EH. 22q11.2 Deletion Syndrome. In: Adam MP, Ardinger HH, Pagon RA, Wallace SE, Bean LJH, Mirzaa G, et al., editors. GeneReviews((R)). Seattle (WA): University of Washington; 1993.
